# Fifty years of gating currents and channel gating

**DOI:** 10.1085/jgp.202313380

**Published:** 2023-07-06

**Authors:** Luigi Catacuzzeno, Franco Conti, Fabio Franciolini

**Affiliations:** 1 Department of Chemistry Biology and Biotechnology, University of Perugia, Perugia, Italy; 2 Department of Physics, University of Genova, Genova, Italy

## Abstract

We celebrate this year the 50th anniversary of the first electrophysiological recordings of the gating currents from voltage-dependent ion channels done in 1973. This retrospective tries to illustrate the context knowledge on channel gating and the impact gating-current recording had then, and how it continued to clarify concepts, elaborate new ideas, and steer the scientific debate in these 50 years. The notion of gating particles and gating currents was first put forward by Hodgkin and Huxley in 1952 as a necessary assumption for interpreting the voltage dependence of the Na and K conductances of the action potential. 20 years later, gating currents were actually recorded, and over the following decades have represented the most direct means of tracing the movement of the gating charges and gaining insights into the mechanisms of channel gating. Most work in the early years was focused on the gating currents from the Na and K channels as found in the squid giant axon. With channel cloning and expression on heterologous systems, other channels as well as voltage-dependent enzymes were investigated. Other approaches were also introduced (cysteine mutagenesis and labeling, site-directed fluorometry, cryo-EM crystallography, and molecular dynamics [MD] modeling) to provide an integrated and coherent view of voltage-dependent gating in biological macromolecules. The layout of this retrospective reflects the past 50 years of investigations on gating currents, first addressing studies done on Na and K channels and then on other voltage-gated channels and non-channel structures. The review closes with a brief overview of how the gating-charge/voltage-sensor movements are translated into pore opening and the pathologies associated with mutations targeting the structures involved with the gating currents.

## Teaser

Retrospective on gating currents from their first recording in 1973 to the present day and their contribution to understanding channel gating.

## Hodgkin and Huxley’s contribution to the gating currents

This year we celebrate the 50th anniversary of the first electrophysiological recordings of the gating currents from voltage-gated ion channels reported in 1973 by Schneider and Chandler (1973) for Ca channels of skeletal muscle and by Armstrong and Bezanilla (1973) for the Na channel of the squid giant axon. This retrospective is dedicated to those events and what followed in the next 50 years. The narrative must however begin 20 years earlier, with the publication in 1952 of a series of papers by Hodgkin and Huxley in which they described the Na and K currents underlying the action potential, and envisioned the charged gating particles that need to move across the membrane to switch them on and off, and the gating currents that their movement would generate (Hodgkin and Huxley, 1952a, 1952b, 1952c; Hodgkin et al., 1952).

### Hodgkin and Huxley postulated the “charged gating particles”

**Figure 1.**
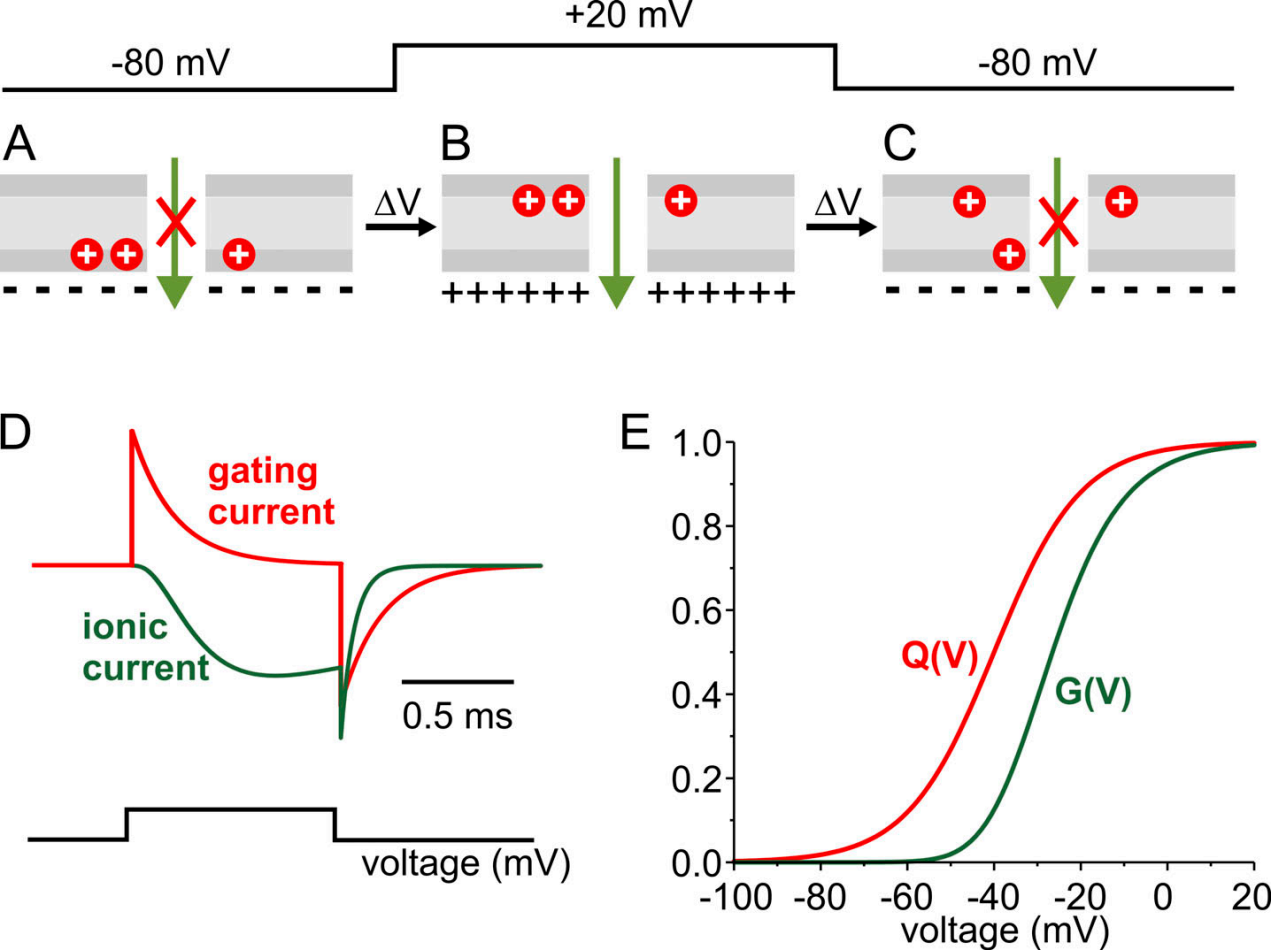
**Hodgkin and Huxley’s charged gating particles. (A–C)** Sketch illustrating the membrane relocation of the three-activating charged gating particles of Na channel upon membrane depolarization and repolarization. **(D)** Time courses of gating particles’ translocation (red trace) and Na currents (green trace) obtained using Eqs. 5 and 6 of Box 1 for the activation phase, and Eqs. 7 and 8 for the deactivation phase, respectively, considering three activation particles and one inactivation particle. **(E)** Theoretical voltage dependence of charged gating particles translocation, Q(V), and channel conductance, G(V), for a voltage-gated ion channel, obtained using Eqs. 9 and 10 of Box 1, respectively.

Hodgkin and Huxley found that both Na and K currents rose on applying a depolarizing step from negative potentials and fell back to zero on repolarization. The observation that these currents were strictly controlled by the membrane potential led them to postulate the presence of charged particles that move across the membrane in response to voltage changes and control the gating for Na and K currents (Fig. 1, A–C; see also Box 1). They further noticed that on applying depolarizing steps, both currents (more rigorously, their conductances, g_Na_ and g_K_) rose following a sigmoid time course, whereas on repolarization they fell exponentially toward zero, and much faster than they rose. These observations made them think that g_Na_ and g_K_ were each controlled by a number of independent gating particles (three for g_Na_ and four for g_K_) in a way that all of them had to be in the “permissive” position for the membrane to pass Na^+^ and K^+^ ions (Fig. 1 B for g_Na_), but only one of them would suffice to switch back to the “non-permissive” position to make the membrane impermeant again to Na^+^ and K^+^ ions (Fig. 1 C, still for g_Na_).

For a satisfactory fit of the sigmoid time course of activation of g_Na_ and g_K_, the model parameters required the translocation of three gating particles for g_Na_ (*m* = 3) and four for g_K_ (*n* = 4). Hodgkin and Huxley were well aware that more particles would fit the data just as well, if not better, but they judged the improvement was not worth the effort. In any event, these values for *m* and *n* found by Hodgkin and Huxley represented a remarkable prediction of what came next. Four *n* particles for g_K_ matches the four subunits that make up typical voltage-gated K channels, while three *m* particles for g_Na_ can be correlated to domains I through III, which we now know to control the activation process in voltage-gated Na channels (while domain IV mainly appears to control inactivation). It may be useful to recall that at the time Hodgkin and Huxley proposed the charged gating particles, there was no concept of ion channels as we know them today, and in fact they never used the word “channel” in their 1952 papers.

The postulated gating particles were assumed to be electrical charges or dipoles that relocate in the membrane in a voltage-dependent manner. At negative voltages, typical of resting excitable cells, the postulated positively charged particles would be positioned toward the intracellular side of the membrane, stabilized by the excess negative countercharges inside. In this position, the membrane would not pass ions. Upon depolarization, the charged particles would move outward, and this very movement would increase the membrane permeability to Na^+^ and K^+^ ions (Fig. 1, A–C).

An important aspect of the Hodgkin and Huxley voltage sensor hypothesis was that the time course of the postulated gating charged particles’ relocation across the membrane following a membrane depolarization could be easily predicted (Catacuzzeno and Franciolini, 2022). Viewed in the simplest form, the movement of the gating particles across the membrane could be pictured as a two-state process, with the gating particles residing in either a non-permissive (*N*) position toward the internal side of the membrane, attracted by the negative resting potential, or pushed on the external side in a permissive position (*P*), upon membrane depolarization.

N↔P. (1)

The forward and backward transition rates, $k_{N\to P}$ and $k_{P\to N}$, would govern the process and are expected to have a voltage dependence of the form

$$k_{N\to P}=k_{N\to P}\left(0mV\right)e^{ze_{0}\gamma V/kT}andk_{P\to N}=k_{P\to N}\left(0mV\right)e^{-ze_{0}\delta V/kT}\text{,}$$ (2)

where *z* is the charge carried by the gating particle and *γ* and *δ* are the fractions of the voltage drop experienced by the particle during its movement in the forward and backward direction, respectively.

**Scheme 1.**
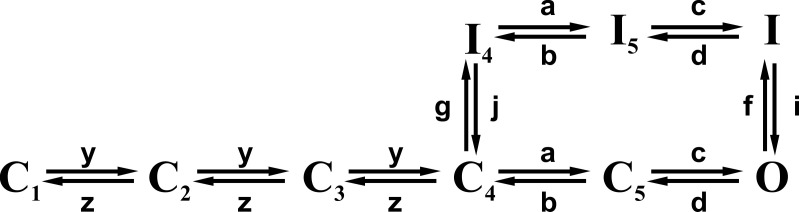
**Kinetic scheme of Na channel gating.** The kinetic scheme shows that inactivation can be reached from a closed state as well as from the open state (from Vandenberg and Bezanilla, 1991a).

According to the kinetic Scheme 1, the time course of the fraction of particles in a permissive position (*f*_*P*_) will be governed by the differential equation

$$\frac{df_{P}}{dt}=k_{N\to P}\left(1-f_{P}\right)-k_{P\to N}f_{P}\text{,}$$ (3)

having the formal solution

$$f_{P}=f_{P\propto }-\left(f_{P\propto }-f_{Pi}\right)e^{-\left(k_{P\to N}+k_{N\to P}\right)t}\text{,}$$ (4)

where *f*_*Pi*_ and $f_{P\infty }=k_{N\to P}/\left(k_{N\to P}+k_{P\to N}\right)$ are the initial and steady-state fractions of permissive particles, respectively.

Assuming to apply a depolarizing step from a very negative potential (*f*_*Pi*_≈0) and that three particles are needed to be in the permissive position to open the channel, the activation time course ion conductance will be proportional to the third power of *f*_*P*_:

$$I\propto f_{P}^{3}=f_{P\propto }^{3}{\left[1-e^{-\left(k_{P\to N}+k_{N\to P}\right)t}\right]}^{3}\text{,}$$ (5)

while the current carried by the moving gating particles, *I*_*gp*_, can be described by the rate of change of the fraction of gating particles in the permissive position

$$I_{gp}=z\left(\gamma +\delta \right)e_{0}\frac{df_{P}}{dt}=z\left(\gamma +\delta \right)e_{0}f_{P\propto }\left(k_{P\to N}+k_{N\to P}\right)e^{-\left(k_{P\to N}+k_{N\to P}\right)t}\text{.}$$ (6)

On this base, the time course of *I*_*gp*_ in response to a depolarizing step would feature an instantaneous rise followed by a monoexponential decay (Eq. 6), while the ion current is expected to have a sigmoidal rise (Eq. 5; Fig. 1 D).

Conversely, on repolarization we will have *f*_*Pi*_≠0 and $f_{P\propto }\approx 0$, which would give

$$I\propto f_{Pi}^{3}e^{-3\left(k_{P\to N}+k_{N\to P}\right)t}\text{,}$$ (7)

$$I_{gp}=z\left(\gamma +\delta \right)e_{0}\frac{df_{P}}{dt}=-z\left(\gamma +\delta \right)e_{0}f_{Pi}\left(k_{P\to N}+k_{N\to P}\right)e^{-\left(k_{P\to N}+k_{N\to P}\right)t}\text{.}$$ (8)

According to Eqs. 7 and 8 and on the notion that the return of only one gating particle to the non-permissive position is sufficient to stop the current, upon repolarization the decay phase of *I*_*gp*_ will have a time course three times slower than the decay of the ion current (Fig. 1 D). Both currents will instead decay following a single exponential time course (Fig. 1 D).

As for the steady-state voltage dependence of the charged gating particles, translocation, Q(V), and membrane conductance, G(V), from Eqs. 2 and 3, we will have the following Boltzmann-like forms

$$Q=\int_{0}^{\infty }I_{g}dt=z\left(\gamma +\delta \right)e_{0}f_{P\propto }=\frac{z\left(\gamma +\delta \right)e_{0}}{1+e^{-ze_{0}\left(V-V_{1/2}\right)/kT}}\text{,}$$ (9)

$$\frac{G}{G_{\max}}={\left[\frac{1}{1+e^{-ze_{0}\left(\gamma +\delta \right)\left(V-V_{1/2}\right)/kT}}\right]}^{3}\text{.}$$ (10)

Notice that the Q(V) relationship has the form of the classic Boltzmann curve, while the G(V) relationship will be given by the third power of the Q(V) Boltzmann relationship (Fig. 1 E). This is so on the grounds that three gating particles are needed to open one permeating pore in the membrane. This occurrence results in the G(V) curve being significantly shifted toward more depolarized potentials compared with the Q(V) curve (Fig. 1 E).

### Hodgkin and Huxley envisioned the gating currents

Hodgkin and Huxley further postulated that the charged gating particles moving across the membrane upon voltage changes should generate very small currents that ought to develop before the ion currents are activated. In principle, there was no alternative to this conclusion as Clay Armstrong recounted years after the postulated gating currents were observed (Armstrong, 1981). Hodgkin and Huxley, however, added that these gating currents had to be very small, hardly more than a few percent of the maximal Na current, as they tried to detect them by carrying out experiments at the Na equilibrium potential where no Na current would be present, but their efforts were in vain. When the Na gating currents were finally recorded, 20 years later, they were in fact about 2% of maximum Na current.

Because the gating currents are the readout of the gating particles’ translocation across the membrane following changes of the electric field, they should share the features of capacitive currents as instantaneous rise and exponential decay. A second prediction, again based on the assumption that these are in principle capacitive currents, was that the amount of charge moving toward the external side of the membrane following a depolarization (the ON gating charge, *Q*_*ON*_) should be the same as that moving back to its original positions following repolarization (the OFF gating charge, *Q*_*OFF*_). We will see that neither expectation was met when the gating currents were recorded.

## Gating current recordings from native Na and K channels

Following Hodgkin and Huxley’s prediction of the gating currents, several groups set out to record them. The task was demanding due to their small size and fast kinetics, and because other bigger currents would activate in the first few milliseconds of the voltage step, the time frame of the gating currents development. For these reasons, the first recordings of the gating currents, reported in 1973, initially in connection with excitation–contraction coupling in the skeletal muscle (Schneider and Chandler, 1973) and a few months later in the squid axon (Armstrong and Bezanilla, 1973), had to wait more than 20 years from their initial prediction.

### First gating current recordings

Using frog skeletal muscle fibers, Schneider and Chandler (1973) were the first to report gating currents (nonlinear charge translocation) that rose fairly rapidly but decayed very slowly. They recognized that their gating currents not only had several features in common with those recently obtained by Armstrong and Bezanilla for the Na channel of the squid axon (their *Nature* paper reporting them was to be published only a few months after Schneider and Chandler’s) but also showed major differences: the much bigger charge translocated per membrane unit area and the much slower decay rate. On this evidence, they interpreted the gating currents as derived from the translocation of the voltage sensors on the T-tubule membrane involved in the excitation–contraction process (Schneider and Chandler, 1973).

On the squid giant axon, Armstrong and Bezanilla, who were trying to record gating currents from Na channels, had to cope with two major types of current that superposed on the gating currents, the Na ion current and the capacitive current needed to charge the membrane in the voltage-clamp experiments. The Na current was easily removed by replacing permeant Na^+^ ions with impermeant ones (Tris or NMDG) and making use of toxins to block their conducting pathways (i.e., tetrodotoxin). That was what Armstrong and Bezanilla did when they first, in 1971, unsuccessfully tried to record the gating currents from the squid giant axon. The complication was, as they were aware of, the large linear capacitive current needed to charge the membrane to the new voltages, which in principle had a time course similar to the voltage-dependent “capacitive” gating current.

**Figure 2.**
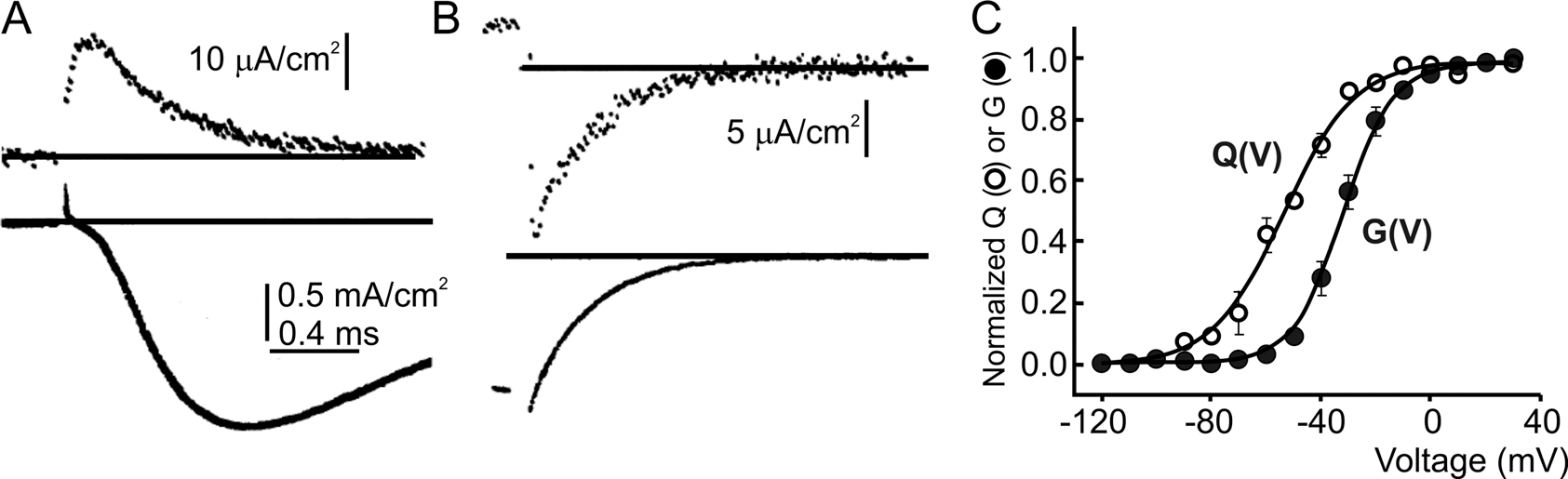
**Gating current recordings. (A and B)** Turn-on and turn-off of Na gating currents (top) and Na ion current (bottom) in response to a depolarizing step to 0 mV. The gating currents and the ion currents are from different axons (from Armstrong and Bezanilla, 1973). **(C)** Relation of the normalized charge vs. potential (Q(V)) and conductance vs. potential (G(V)) for the bacterial Na channel, NaChBac (from Kuzmenkin et al., 2004).

On these grounds, the following summer at Woods Hole they set out to record the gating currents using a stimulation protocol that alternated positive and negative pulses—initially of equal size (the ±P protocol), then scaling down fourfold the negative pulse (the P/−4 protocol)—to eliminate by subtraction the linear components of the capacity transients. With this protocol and some improvements of the electronics, they were able to record gating currents from the squid giant axon. The currents activated very rapidly upon depolarization, decayed rapidly, and were essentially over before any significant Na ion current was activated (Armstrong and Bezanilla, 1973; Fig. 2 A). In the following year, transient currents in the squid axon were reported by Keynes and Rojas (1974) at the Marine Station in Plymouth, and soon after by several others on different cell models (e.g., Meves, 1974; Nonner et al., 1975).

Because of their postulated behavior as capacitive currents, the gating currents were expected to rise instantaneously and decay with a single exponential time course. As we will see, neither of these expectations was to be met.

#### The ON gating current

Upon recording gating currents, it was soon observed that if for small depolarizations (around 0 mV) their rise was essentially instantaneous, as expected from the Hodgkin and Huxley’s two-state model, at higher depolarizations (∼50 mV and more) it was not, showing a clear rising phase that could last several tens of microseconds. This could be shortened by applying stronger depolarizations and using faster voltage clamp systems, but not eliminated. This led Armstrong and Bezanilla to think that it was an essential feature of the gating currents and not an artifact of the subtraction procedure used in the gating currents recording/analysis, or resulting from slow-voltage clamping (Armstrong and Bezanilla, 1977; Bezanilla and Taylor, 1978; Armstrong and Gilly, 1979). They suggested that the rising phase could be the result of slow early transitions in channel gating (compared to later transitions), less voltage-dependent, or carrying less charge (Armstrong and Gilly, 1979; Taylor and Bezanilla, 1983). Years later Bezanilla and coworkers reexamined the question having noticed a distributed series resistance and non-linear capacitance charging due to the complex wrapping of the squid giant axon membrane by the Schwann cells and connective tissue. By adopting maneuvers that would allow charging the membrane with a single time constant, they found that the observed rising phase of the gating currents virtually disappeared, concluding that it was an artifact due to poorly compensated series resistance in the Frankenhaeuser–Hodgkin space of the squid giant axon, leading to inhomogeneous charging of the axon membrane (Stimers et al., 1987).

Genuine gating current rising phases continued, however, to be seen in the squid giant axon at high positive potentials, provided special care was taken at compensating the series resistance (Keynes et al., 1990), as well as in several other axonal preparations, such as crayfish, different *Loligo* species, and the frog node of Ranvier (Armstrong and Gilly, 1979; Meves and Pohl, 1990; Starkus and Rayner, 1991; Ichikawa et al., 1991). Although the evidence for the rising phase was very different in the various studies and rarely prominent, the occurrence of being reported in so many instances came to be considered a specific feature of the ON gating current of the Na channel, although of marginal relevance. We recall that a clear rising phase was later found by Bezanilla et al. (1991) for K gating currents from expressed Shaker channels. Of note, the experimental observation of a genuine rising phase in the ON gating current, not contemplated in the original model of Hodgkin and Huxley, was the first evidence that the voltage sensors move along a sequence of energetically stable intermediate states before assuming the fully activated position.

As for the decay phase of the ON gating current of the Na channel, Armstrong and Bezanilla found that it was not described by a single exponential, as expected from the two-state model of Hodgkin and Huxley’s gating particles, but displayed at least two major components (Armstrong and Bezanilla, 1974). While the faster component could be arguably associated with the relaxation of the gating charges, the slower component was more difficult to interpret. It was initially thought to represent the movement of charges associated with inactivation (i.e., the movement of the inactivation particle, *h*, in Hodgkin and Huxley’s model), although it seemed to develop significantly faster. Internal perfusion of the axon with pronase, the proteolytic enzyme that removes inactivation of the Na current (Armstrong and Bezanilla, 1973), showed that while inactivation was greatly decreased, the slow decay component of the gating current was not visibly affected (Armstrong and Bezanilla, 1977), consistent with the idea that it was not associated with the movement of the inactivation particle of Hodgkin and Huxley’s model. Later fluorescent labeling experiments, to be described below, showed that the slow component was in fact not associated with inactivation, but part of the activation process (Chanda and Bezanilla, 2002).

#### The OFF gating current

Another discordant feature with Hodgkin and Huxley’s classic view was found in the time course of the OFF gating current. According to their model, while all three independent gating particles needed to be in the permissive position for the Na channel to open (in modern terms), only one single particle was sufficient to switch back to the non-permissive position for the channel to close (as shown in Fig. 1 C). Gating currents, by contrast, were contributed—the ON and OFF currents alike—by all three particles. In other words, the OFF gating current would depend on *m* kinetics while the Na ion currents on *m*^*3*^ kinetics, with the result that the Na ion current should be expected to have a deactivation decay three times faster than the decay of the OFF gating current. Experimental data showed instead that the OFF gating current decay was only slightly slower than the Na current deactivation (rarely exceeding 1.2 times; Armstrong and Bezanilla, 1974; Bezanilla and Armstrong, 1975), as illustrated in Fig. 2 B showing for comparison the OFF gating current (top) and the deactivation Na current (bottom). These data show that the return of the gating charges to their resting position is a more complex process than initially thought. However, this behavior could be reproduced by dismissing the assumption that gating particles move independently (Vandenberg and Bezanilla, 1991a), as described below.

Fig. 2 C reports the experimental results of the voltage dependence of the gating charge translocation, Q(V), and the channel conductance, G(V), for the bacterial Na channel, NaChBac (Kuzmenkin et al., 2004). Note the great similarity with the theoretical expectations illustrated in Fig. 1 E.

### Na gating current immobilization and channel inactivation

**Figure 3.**
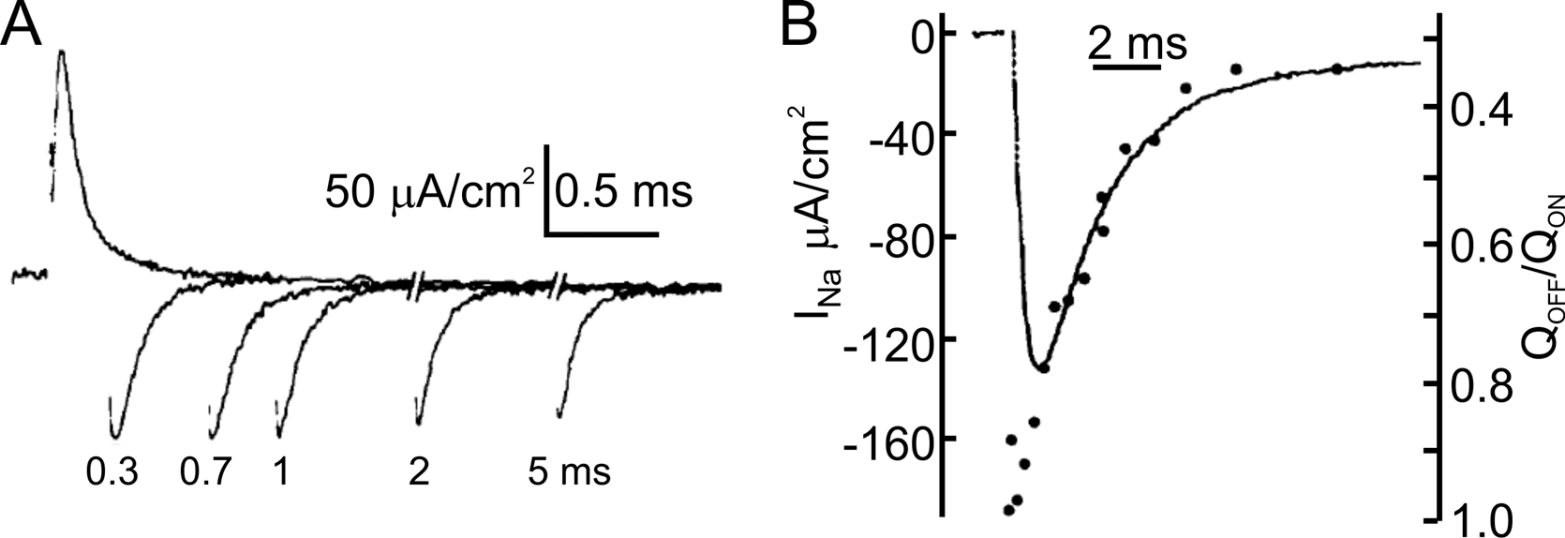
**Immobilization of the gating charges. (A)** The OFF-gating charge of the Na channel gets immobilized with long pulses. **(B)** The OFF-gating charge of the Na channel gets immobilized (circles) with long pulses in parallel with channel inactivation (line) (from Armstrong and Bezanilla, 1977).

Another observation at variance with the very notion of gating currents as the capacitive movement of charged particles was the different amount of the ON and OFF gating charge translocated (measured as their time integrals, *Q*_*ON*_ and *Q*_*OFF*_) that was observed with long pulses (Fig. 3 A). Namely, the Na *Q*_*OFF*_ was generally found to be of similar size as the *Q*_*ON*_ for short (<1.0 ms) depolarizing pulses, but to decrease with depolarization length and stabilize at around one-third of *Q*_*ON*_ for pulses longer than 10 ms (circles in Fig. 3 B). Notably, this charge immobilization (the time-dependent decrease of *Q*_*ON*_*/Q*_*OFF*_ ratio) paralleled the time course of Na current inactivation (solid line of Fig. 3 B), as assessed at different voltages. These results suggested that the development of inactivation immobilized a significant fraction of the gating charges which were no longer capable of regaining their initial positions upon repolarization. In line with this view, pronase, known to remove Na current inactivation, decreased charge immobilization in parallel by nearly corresponding amounts (Armstrong and Bezanilla, 1977), whereas agents like local anesthetics that enhance inactivation promoted charge immobilization (Yeh and Armstrong, 1978). Further evidence of stringent coupling between Na channel inactivation and gating charge immobilization was that recovery of charge immobilization followed the same time course as the recovery from inactivation (Armstrong and Bezanilla, 1977; Nonner, 1980).

The results of Armstrong and Bezanilla (1977) on Na gating charge immobilization had two important consequences beyond the field of gating current. First, they showed that the inactivation of the Na channel was not the result of independent gating particles that moved in a voltage-dependent manner across the membrane, as Hodgkin and Huxley’s model suggested. On the contrary, inactivation was strictly linked to channel activation, and its voltage dependence was not intrinsic to the inactivation process, but the result of its coupling with the voltage-dependent activation. Second, it was upon observing the effect of pronase in removing inactivation and charge immobilization in parallel that Armstrong and Bezanilla proposed the ball-and-chain model of Na channel inactivation, whereby a portion of the channel protein has access to the pore when it is in the open state and blocks it (Armstrong and Bezanilla, 1977). Based on this view, the interaction of the ball with its receptor could be the step that originates the immobilization of the gating charges associated with channel inactivation.

#### Gating models of the Na channel helped to interpret gating charge immobilization and OFF gating currents time course

Gating models of the Na channel from the early 1980s suggested already that the channel could enter the inactivated state also from closed states (Aldrich et al., 1983; Horn and Vandenberg, 1984), but it was the simultaneous recording of single-channel currents from the squid giant axon together with the ion and gating currents (Bezanilla, 1987) that allowed more defined channel gating schemes to be drawn. These new schemes all included several closed states and inactivated states that could be reached from open states as well as the closed states, as illustrated in the representative model below (Scheme 1; Vandenberg and Bezanilla, 1991a), which represents a modification of the scheme developed by Armstrong and Bezanilla (1977). Notably, these inactivated states were supposedly those that immobilize the gating charge.

In this model, the forward rate constants connecting states C1 to C4 all have the same values, as do the corresponding backward rate constants, as they best fitted the data. Notably, this linear gating model for Na channels, with equal forward rate constants and equal backward rate constants (which makes the three gating particles no longer independent in their motion), has the relevant property that the ratio of the deactivation time constants of the gating current to the ion current is not 3 as expected from Hodgkin and Huxley’s independent gating particles model (with forward rates 3α, 2α, and α, and backward rates β, 2β, and 3β), but close to 1.2, as found experimentally (Fig. 2 B; Vandenberg and Bezanilla, 1991a).

The gating charge translocated at each step of the proposed scheme was estimated from the voltage dependence of the connecting rates. The resulting values were 1.5e_0_ for the first three transitions, C1→C2, C2→C3, and C3→C4, 0.4e_0_ for the transition C4→C5, and 1.9e_0_ for opening the channel (transition C5→O). No charge transition was associated with the translocations involving inactivated states. Adding up, the overall charge translocated to open a channel from the deepest closed state is 6.8e_0_. This value is only about half of what was later found (12–14e_0_; Yang et al., 1996; DeCaen et al., 2011)^1^. The reason for this low estimate is not clear.

### Gating current recordings from native K channels

Early attempts to record gating currents from K channels had to consider that their kinetics could be especially slow, given that the ion K currents were in general about 10-fold slower than Na currents and a similar ratio could be arguably assumed to hold for the kinetics of their gating currents. This was thought to be the reason why the K gating currents were not seen when recording the Na gating currents with experiments usually done at a low temperature (about 5°C) to slow the fast Na gating currents kinetics. On this ground, the first attempts made by Bezanilla and coworkers on the squid giant axon were carried out at room temperature to make the K gating current emerge from baseline and the Na gating current develop so fast to go essentially unseen (Bezanilla et al., 1982).

Other actions were taken by Bezanilla and coworkers to further reduce the still potentially contaminating Na gating currents. They used rather depolarized holding potentials to inactivate Na channels and immobilize a good fraction of their gating charges, or applied internal local anesthetics and replaced external Cl^−^ with NO_3_^−^, two maneuvers that each individually inhibited both the Na ion and gating currents to a great extent (White and Bezanilla, 1985). These actions allowed them to record putative K gating currents which had kinetics congruent with the ion K currents. K gating currents could be seen more neatly when negative prepulses were applied so as to delay their activation and thus separate them better from the residual Na gating currents (White and Bezanilla, 1985). The improved separation between the two gating current types disclosed a clear rising phase on the K gating currents, which could arguably represent a genuine feature of the current, given the much slower kinetics and thus the unlikely presence of artifacts due to fast kinetics.

## Cloning of Na and K channels, the sliding helix model, and the gating currents from expressed channels

### The primary structure of a Na channel and the sliding helix model

In 1984, the voltage-gated Na channel from *Electrophorus electricus* electroplax was cloned and its amino acidic sequence was elucidated (Noda et al., 1984). Based on the hydropathy plot, the ∼1,800-residue polypeptide chain would fold to form four homologous domains (I–IV), each predicted to contain six transmembrane α-helical segments (S1–S6). Of these, the S4 segment of each domain was further shown to contain a high number (4–7) of positive charges (usually arginine), systematically interposed by two non-charged residues. This unexpected concentration of positively charged residues in the S4 segments immediately suggested that it was the long sought-after voltage sensor of the Na channel (Noda et al., 1984; Greenblatt et al., 1985).

**Figure 4.**
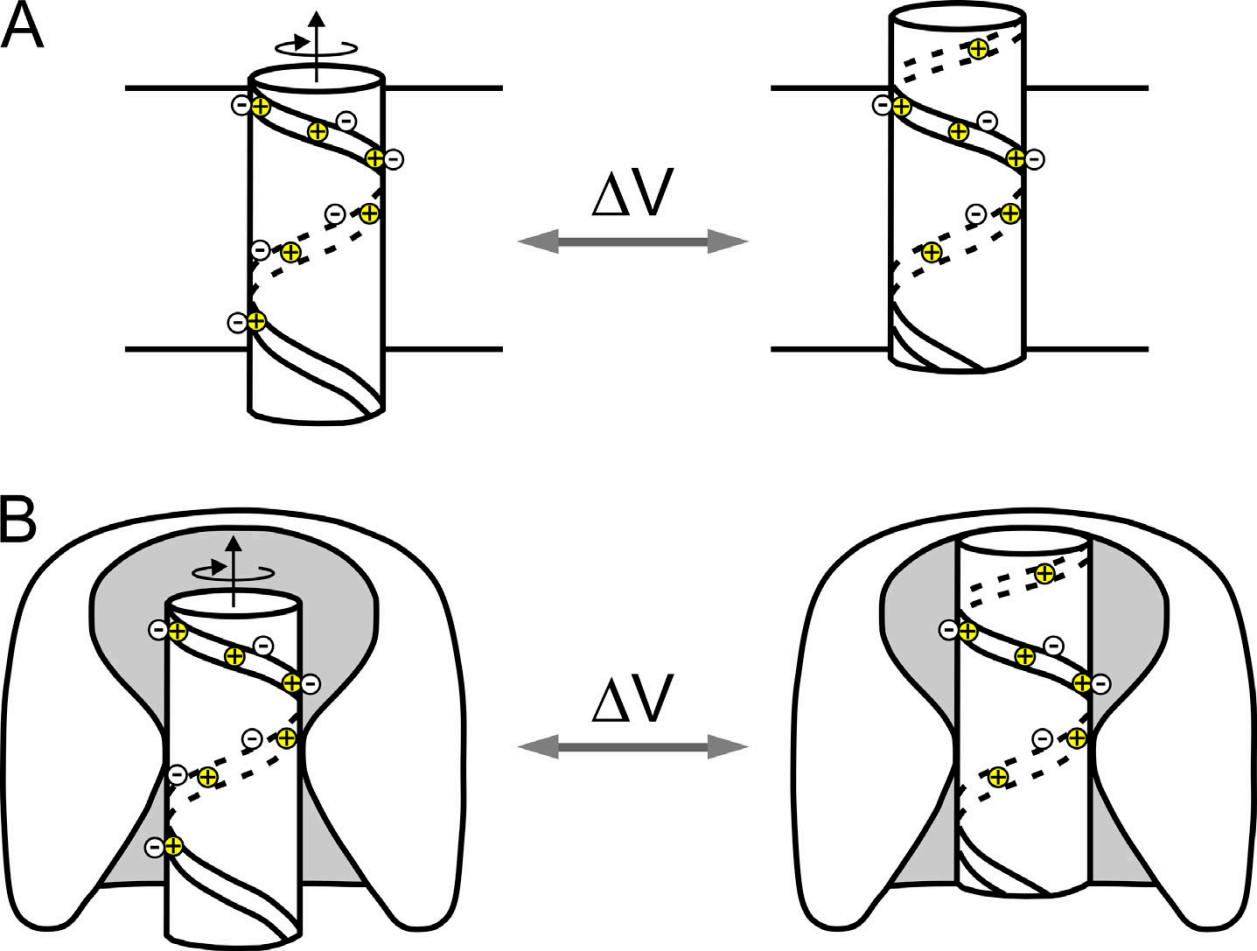
**The sliding helix model. (A)** Schematic of the sliding helix or helical screw model of channel gating as proposed by Catterall (1986) and Guy and Seetharamulu (1986), suggesting that the positive charges (mostly arginine), arranged in a spiral shape, pull the S4 segment inward at the negative potential of the resting state. Upon depolarization, the inward-directed forces are released and the S4 segments are pushed outwards following a spiral path which allows the positive charges to pair in succession with the negatively charged residues on neighboring transmembrane segments (from Catterall, 1986). **(B)** Evolution of the sliding-helix model to include the focused electric field and the water-filled vestibules on the extracellular and intracellular sides (modified from Catterall, 2000).

In light of these new data, Catterall (1986) and separately Guy and Seetharamulu (1986) reached, with their respective “sliding helix” and “helical screw” models, similar mechanistic conclusions for the S4/voltage sensor motion. They both proposed that in the resting state (negative potential inside), the positive charges on the S4 α-helix were pulled inward by electrostatic forces (Fig. 4, A and B, left). In this position they would interact with negatively charged residues on neighboring helices, forming ion pairs that stabilize the S4 segment in an apparent hydrophobic environment. Upon membrane depolarization and the release of these inward-directed forces, the S4 segments would move outward in a spiral movement (Fig. 4, A and B, right) that would let positive charges step up and pair in succession with the negatively charged residues on neighboring transmembrane domains (S1–S3 segments). With regards to the sliding helix model, it should be mentioned that Clay Armstrong, without any structural information on ion channels, proposed a similar model in 1981, in which he suggested the pairing of positive and negative gating charges on different mobile components of the channel, and small ratcheting motions that would move every partner one step and transfer one whole charge across the membrane (Armstrong, 1981).

The primary structures of the Shaker K channel from *Drosophila* (Tempel et al., 1987; Kamb et al., 1988; Pongs et al., 1988) and Ca channels from various muscle types (Tanabe et al., 1987; Mikami et al., 1989; Koch et al., 1990) were released shortly afterward and found to share the same architecture of Na channels, with special reference to the S4 segment, further reinforcing the view that this was the voltage sensor of voltage-gated channels.

### Testing the sliding helix model

The primary prediction of the sliding helix model was the outward movement of S4 during channel activation. The first evidence of this actually occurring came from cysteine mutagenesis studies on Na channels showing that the S4 segments move outward upon channel activation and translocate with their movement the gating charges from the internal to the external cleft of the voltage-sensor domain (VSD). In particular, the results of Horn and coworkers, who sequentially replaced the eight basic residues in the S4 segment of DIV with cysteine to generate targets for sulfhydryl-modifying reagents, provided evidence that some S4 residues could be exposed alternately to the intracellular or extracellular solution in the resting and the activated states and that different positions close together in S4 could be modified from different sides of the membrane (Yang and Horn, 1995; Yang et al., 1996). These results, rather unexpected at the time, provided the first indication that the electric field falls across a narrow part of the VSD and that some charges could cross the entire electric field.

Studies on Shaker channels reached essentially the same conclusions. They showed that cysteines at positions 362 and 365, in replacement of arginine, were only accessible from the external VSD water cleft at depolarized voltages, while the cysteine at position 368 was accessible from the internal cleft at hyperpolarized voltages (Larsson et al., 1996). These results, suggesting that depolarization pushes the S4 segment upward, gained support from voltage clamp fluorometry experiments on Shaker channels that traced the movement of individual residues on S4 upon voltage changes (Cha and Bezanilla, 1998; Cha et al., 1999) and from histidine scanning mutagenesis experiments that label gating charge locations and probe their accessibility to protons (Starace and Bezanilla, 2001; Starace and Bezanilla, 2004; Chanda and Bezanilla, 2008).

We recall here that early crystal structures of several voltage-gated channels (NavAb, NaChBac, NaV1.4, and Kv1.2) showed that portions of the S4 segment were found in a 3.10-helical conformation (Chen et al., 2010; Payandeh et al., 2012; Zhang et al., 2012; Pan et al., 2018), as functional data using histidine scanning on S4 segment and metal ion bridges also suggested (Villalba-Galea et al., 2008; Henrion et al., 2012). Although various studies indicated 3.10-helical conformations to involve different portions of the S4 segment of varying lengths, the prevalent view was that a short running portion of α-helical S4 converts transiently—and this is important for the proper functioning of the system—into a 3.10-helical conformation when the sensor passes through the central constriction of the VSD during activation. A view that found further support from a recent study using Zn^2+^ ion bridges and disulfide bonds shows that inhibition of α-helical to 3.10-helical conversion inhibits channel activation (Bassetto et al., 2020).

#### Testing the contribution of S4 charges to channel gating

The second prediction of the sliding helix model is that the charges on S4 are instrumental to channel gating. To test this, Conti, Stühmer, and coworkers assessed the voltage sensitivity changes of expressed Na channels when one or more positive charges on S4 of domain I were replaced by neutral or negative residues. They found that increasingly reducing the overall net positive charge on S4 resulted in a decreasing trend of the apparent gating charge *z*_*g*_ and a rightward shift of the voltage dependence (Stühmer et al., 1989). Although not conclusive (because several mutants were difficult to express and others displayed deteriorated gating properties), their study provided a first experimental evidence that S4 was the voltage sensor of the Na channel.

Studies with a similar approach were repeated a few years later on the expressed Shaker channel, which confirmed the earlier results on voltage-gated Na channels (Logothetis et al., 1993). They found, however, that the effects of the various mutations were not fully congruent with the canonical view that the charged residues on S4 were functionally equivalent, regardless of their position, as neutralizations of different basic residues on S4 had different effects. These results certainly provided further evidence of the role of the positive charges on S4 in channel activation but also showed that the channel’s voltage dependence could not be fully explained by solely electrostatic considerations.

The contribution of each putative single gating charge on S4 to the total charge, upon full channel activation, was readdressed by Aggarwal and MacKinnon (1996) and Bezanilla and coworkers (Seoh et al., 1996). The two groups estimated the gating charge per channel from the gating currents, factorized for the number of channels estimated with the radioactive channel blocker agitoxin (Aggarwal and MacKinnon, 1996) or with noise analysis in the same patch (Seoh et al., 1996). Bezanilla and coworkers made estimates of the total charge per channel also using a modified limiting slope method. Taking both studies cumulatively, the main observation is that only the first four basic residues on S4 (R1–R4) are effectively involved in the gating process and in generating the gating currents (in other words, a consistent decrease of the gating charge per channel with respect to control was only observed upon neutralizing either of the first four S4 residues). Aggarwal and MacKinnon estimated the contribution of six of the seven positive charges in S4 as no mutation at position R6 produced functional channels^2^. Charge neutralization of the first four arginine residues (R1–R4) led to a marked decrease in the translocated gating charge (∼4e_0_ each). Charge neutralization of K5 decreased the gating charge by ∼2e_0, while_ that of K7 had no effect. Bezanilla and coworkers also showed that neutralization mutations of the first four charged residues in S4, R1–R4, markedly reduced the gating charge compared with WT. The reduction they observed for each charge neutralization tested ranged between 5e_0_ and 7e_0_, significantly more than the ∼4e_0_ reported by Aggarwal and MacKinnon. By contrast, they found no significant reduction for charge neutralization of K5 (K374Q), possibly because to express this mutant they had to co-mutate residue E293Q in S2 (unlike Aggarwal and MacKinnon, who could neutralize K374 residue with mutation K374S and nicely express the mutant channel). Since neutralization of one charged residue in S4 that crosses the whole transmembrane electric field would in principle result in a reduction of four charges per channel at most, Bezanilla and coworkers concluded that all the mutations tested must exert side effects on channel gating that led to overestimate the contribution of each neutralization (Seoh et al., 1996).

#### Assessing the total gating charge per channel translocated upon full activation

In their studies, Aggarwal and MacKinnon (1996) and Bezanilla and coworkers (Seoh et al., 1996) also estimated the total charge per channel translocated following maximal activation. The estimates with gating currents and radioactive agitoxin method of Aggarwal and MacKinnon (1996) gave a value of 13.6e_0_, only slightly bigger than those obtained by Bezanilla and coworkers (Seoh et al., 1996) with the noise method (12.9e_0_) or the limiting slope method (12.6e_0_). These results are in line with several other studies that estimated the translocated gating charge from the gating currents and found it to fall between 12e_0_ and 14e_0_, with no significant difference on whether the number of channels was estimated with noise analysis or toxin binding (Schoppa et al., 1992; Zagotta et al., 1994b; Noceti et al., 1996; Islas and Sigworth, 1999). The potential bias in estimating gating charge based on gating currents, which do not discriminate between the moving charges effectively functional to channel gating and those that just move under the influence of the electric field, was certainly negligible. Using the limiting slope method (Almers, 1978) that arguably measures only the gating charge functional to channel gating, values of *z*_*g*_ for Na and K channels were found by several studies to range between 12e_0_ and 16e_0_ (Almers and Armstrong, 1980; Bezanilla and Stefani, 1994; Zagotta et al., 1994b; Hirschberg et al., 1995; but see Ishida et al. [2015], who found a gating charge of 10e_0_ for the Kv1.2 channel).

### Other gating current properties were disclosed from expressed K channels

Expressed K channels have been the workhorse of electrophysiologists for the last three decades due to their subunit composition, thus smaller size and easier handling, especially when mutation procedures were involved. Gating currents from K channels were mostly studied from channels encoded by the *Drosophila *Shaker gene expressed at high densities in *Xenopus* oocytes. As for the Na gating currents (cf. Fig. 2 C), the voltage dependence of the Shaker Q(V) curve was shifted to more hyperpolarized values as compared with the G(V) curve, indicating that the four sensors must move to the active state before the channel can open. Gating currents from expressed Shaker channels also showed a rising phase, which became more evident on applying hyperpolarizing prepulses (Taylor and Bezanilla, 1983), and gating charge immobilization. Using the cut-open oocyte technique (Stefani and Bezanilla, 1998) and expressed Shaker channels, Bezanilla and coworkers showed that the OFF gating current carried progressively less charge the longer was the pulse, and thus the higher the level of channel inactivation (Bezanilla et al., 1991; Perozo et al., 1992), very much like immobilization of the Na channel in the squid giant axon. Notably, the removal of the N-terminal region, which removes ion current inactivation (Hoshi et al., 1990), fully removed the OFF current immobilization (Perozo et al., 1992), confirming the strict link between channel inactivation and gating charge immobilization, and indicating in the N-terminal domain the module involved in these processes. At around the same time, Heinemann and coworkers reported, however, that the gating charge immobilization measured on chimeric Shaker channels expressed in *Xenopus* oocytes had no strict correlation with inactivation as it was found to occur also for channels that did not inactivate (Stühmer et al., 1991).

Investigation on expressed Shaker K channels crucially contributed to providing a credible interpretation of another feature of the gating current already observed both in Na and K channels at high bandwidth recordings (Keynes et al., 1990; Forster and Greeff, 1992; Sigg et al., 1999) that state rate models had not been able to predict: the fast (few microseconds) spike-like component of the gating current that preceded the rising phase. Using cell-attached macropatches from *Xenopus* oocytes expressing W434F Shaker mutants that are functionally nonconducting (Perozo et al., 1993), together with improved electronics that allowed to voltage clamp the membrane at 200 kHz bandwidth, Bezanilla and coworkers were able to record and study this fast transient current that carried a minimal charge and showed no fluctuations (“noise”; Sigg et al., 2003). They suggested that it was produced by the rapid reequilibration of the gating charges within the energy well of the resting dwell state, in response to the voltage pulse (Sigg et al., 2003). This interpretation has yet no direct experimental support.

### Fluorescent labeling of expressed K channels clears old unexplained results on Na channels

**Figure 5.**
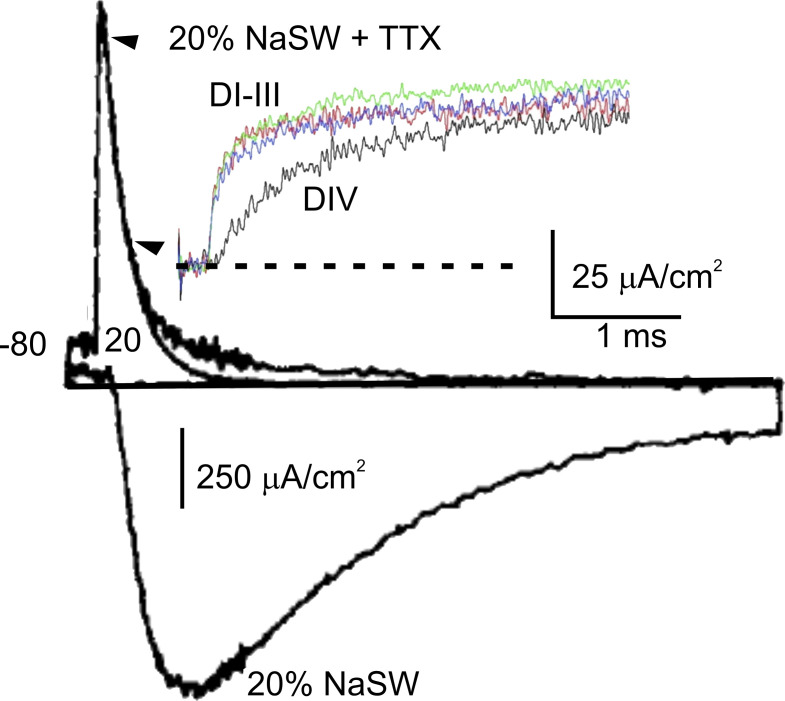
**Native Na gating currents display a fast and a slow decay component.** Fitting the gating current with a single exponential between the two indicated arrowheads uncovers a much slower component that develops over several ms and bore significant charge. Tetrodotoxin (TTX) was added to the artificial sea water (SW) that contained only 20% of natural Na concentration. The ion current, shown by the downward trace, was obtained in a separate experiment on the same axon, under similar conditions as those used for recording the gating current, except for the absence of external TTX. (from Armstrong and Bezanilla, 1977). Inset: Time courses of the fluorescence changes for each of the four S4 segments (from domains I–IV) of the Na channel expressed in *Xenopus* oocytes (from Chanda and Bezanilla, 2002).

By applying site-directed fluorescent labeling to investigate the movement of the four S4 segments of the Na channel during activation, Chanda and Bezanilla (2002) were able to clear another unexplained observation made earlier on Na gating currents: the slow component of the decay phase (Armstrong and Bezanilla, 1974). This slow component that can be seen above the single exponential fitting of the decay time course of the ON Na gating current in Fig. 5 was already shown not to be associated with the inactivation process (Armstrong and Bezanilla, 1977).

Site-directed fluorescent labeling experiments carried out to examine the individual movement of the S4 segments of skeletal muscle Na channels during activation showed that the fluorescence signal of domains I, II, and III rose markedly faster than domain IV (Fig. 5, inset; Chanda and Bezanilla, 2002) and had a time course congruent with the fast component of the gating currents. The rate of change of the fluorescence signal of domains IV was instead compatible with the rate of change of the slow component of the gating current, suggesting that it is likely due to the slow translocation of the S4 segment of domain IV during activation. Notably, when the fluorescence signal of domain IV of the Na channel was recorded simultaneously along with the ion current, the current was observed to rise even before the fluorescence signal had visibly changed, indicating that the activation of domain IV is not required for the channel to activate and conduct (Chanda and Bezanilla, 2002). In this regard, we mention that an earlier study by Horn and coworkers, based on chemical modification of cysteines substituted for residues in the S4 segment of domain IV, suggested that it has major roles in the voltage dependence of slow inactivation (Mitrovic et al., 2000).

### Kinetic models of gating charge translocation

**Scheme 2.**
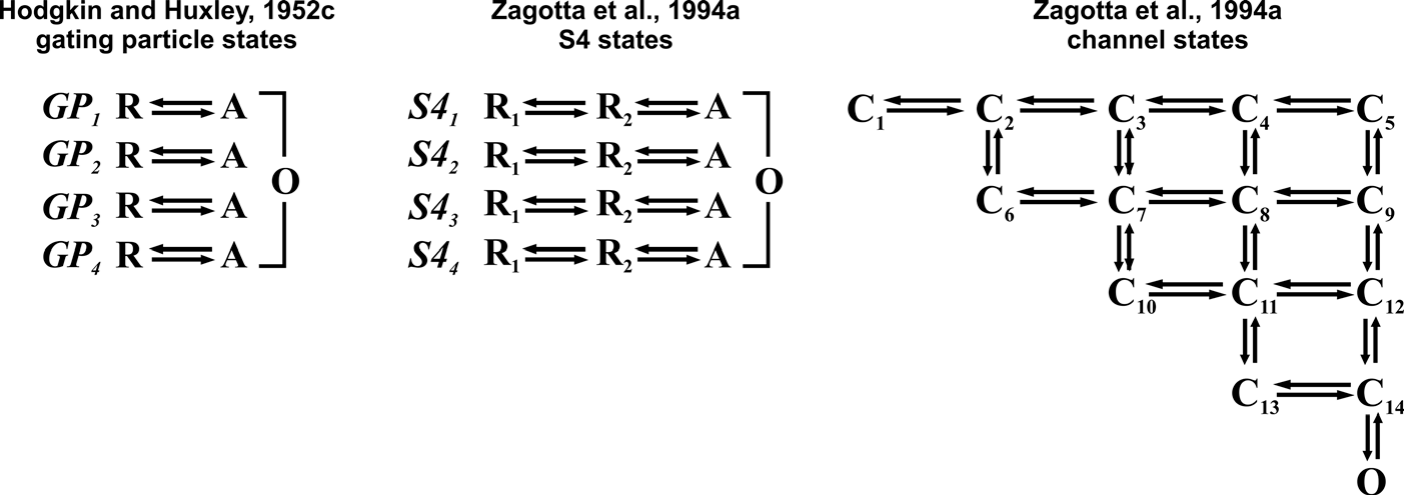
**Kinetic models of gating.** Left: Hodgkin and Huxley’s model of the two-step charged gating particles translocation to open the K channel. GP, gating particles; R, resting; A, activated. Center and right: Gating models of Shaker consistent with a full set of experimental data showing conformational states of each of the four voltage sensors (S4) and the conformational states of the channel, respectively (from Zagotta et al., 1994a).

The first successful model of gating charge translocation during Na and K channels activation was arguably that proposed by Hodgkin and Huxley (1952c), with three and four independent charged gating particles, respectively, each undergoing a single transition from a resting state to an active state, and channels only conducting when all the gating particles are in the active state (Scheme 2, left). Although their model has served greatly as a benchmark and guide for countless subsequent studies, several shortcomings that appeared over time demanded other models more consistent with newer observations to be proposed.

In the 1990s, kinetic models were essentially of two types, parallel and sequential. The elucidation of the tetrameric subunit structure of the Shaker channel (MacKinnon, 1991) steered Aldrich and coworkers toward a parallel model envisioning each voltage sensor (S4) moving independently of each other toward the active state through two conformational changes within each subunit (Scheme 2, center; Zagotta et al., 1994a; Zagotta et al., 1994b). They found instead a high level of cooperativity between the four voltage sensors in the final step that would open the channel, as later confirmed with substitution experiments in the S4 segment by Smith-Maxwell et al. (1998) and Ledwell and Aldrich (1999). The model was constrained to match the large amount of experimental data (both ion and gating currents) the authors had accumulated (Hoshi et al., 1994; Zagotta et al., 1994b). The same model is presented in the extended form on the right of Scheme 2 to show all the kinetic states visited by the channel during full activation.

The model could reproduce very well the major features of the ion currents, but not so well the gating currents. A revisited parallel model was proposed by Schoppa and Sigworth (1998a), who added a second cooperative step after each of the four subunits had reached the active state. This addition was found necessary to account for the two packages of gating charges, ∼1.8e_0_ each, found to be translocated with the cooperative steps leading to channel opening (Schoppa et al., 1992; Schoppa and Sigworth, 1998a).

Sequential models were also used in the last decade of the century. We recall the one proposed by Bezanilla and coworkers, essentially based on Hodgkin and Huxley’s model but enriched with many more states (up to a total of eight). Even not assuming independent translocation of the four voltage sensors, the model could reproduce the experimental data very well in many respects (Bezanilla et al., 1994). In fact, the sequential eight-state model seemed to reproduce several features of the gating currents even better than the parallel three-state model. However, the first crystallographic channel structures that began to appear in the following years, showing the four VSD disposed at the periphery of the channel, far away from each other to make it hard to imagine them functionally connected in any reasonable way, were strongly orienting the scientists’ mood toward parallel and independent models as closer to reality.

In any event, the extensive studies carried out in Aldrich, Sigworth, and Bezanilla’s laboratories over that decade allowed them to reach a congruent view of the kinetics of Shaker channel activation that in its essential elements involved two or three major transitions that occurred independently in each subunit, each carrying a charge close to 1e_0_. A final cooperative transition (possibly two), carrying 1.4–1.8e_0_ per channel, would eventually bring the channel to open (Bezanilla and Stefani, 1994; Bezanilla et al., 1994; Hoshi et al., 1994; Zagotta et al., 1994a, Zagotta et al., 1994b; Schoppa and Sigworth, 1998a; Schoppa and Sigworth, 1998b; Schoppa and Sigworth, 1998c; Ledwell and Aldrich, 1999).

### The gating charge transfer center hypothesis

Cysteine mutagenesis and labeling studies, mainly contributed by Yang and Horn (1995), Larsson et al. (1996), and Yang et al. (1996), and studies by Tiwari-Woodruff et al. (1997) and Tiwari-Woodruff et al. (2000) using the charge reversal mutation method suggested that charged arginine residues R3 (R368) and R4 (R371) on the S4 segment of Shaker interact in succession with the negative aspartate E283 on S2, placed above the hydrophobic plug, during channel activation. X-ray crystal structures of Kv1.2 and Kv1.2/2.1 chimera (Long et al., 2005a; Long et al., 2005b; Long et al., 2007) showed that the arginine residues on S4 also interact with an internal negative charge cluster formed by highly conserved acidic residues (D259 on S3 and E236 on S2 in Kv1.2/2.1 chimera) located right below the hydrophobic plug (Campos et al., 2007). This hydrophobic structure, impermeant to water and ions, separates the intracellular and extracellular water-filled clefts and focuses virtually the whole transmembrane electric field over a distance of 5–10 Å normal to the membrane (Larsson et al., 1996; Yang et al., 1996; Starace and Bezanilla, 2004; Tombola et al., 2005; Chanda et al., 2005). The hydrophobic plug contains a highly conserved phenylalanine residue (at 233 in Kv1.2/2.1 chimera and 290 in Shaker), crucial for gating charge crossing (Bian et al., 2004; Long et al., 2007; Chen et al., 2010).

Based on this structural evidence and the results of their investigation on Shaker channel where they probe several mutations of the conserved phenylalanine at 290 (F290) and the charged residues on S4, MacKinnon and coworkers proposed that the conserved internal negative cluster together with phenylalanine in the hydrophobic plug (F290) form the gating charge transfer center (GCTC), the structure facilitating the sequential translocation of the gating charges across the hydrophobic plug during activation (Tao et al., 2010). Initial models suggesting the cation–π interactions between the arginine guanidinium and phenylalanine as the key contingency that would lower the activation barrier for charge crossing had to be dismissed on the ground that the phenylalanine could be replaced by planar cyclic non-aromatic side chain analogs in the VSD of K and Na channels with no major effects on gating (Tao et al., 2010; Pless et al., 2014).

Demsey and coworkers readdressed the question using MD simulations on the hERG channel modeled on the crystal structure of the Kv1.2/2.1 chimera (Colenso et al., 2014). The hERG channel was chosen because its VSDs contain an extra aspartate on S2, one helical turn above the GCTC, at position 466, so that F463 has two equidistant aspartate residues (D460 and D466), one on each side, and this charge-paired set-up was expected to disclose mechanistic features governing arginine crossings. They found that once arginine is released from the internal negative cluster and moves up under the influence of the electric field, it interacts with phenylalanine F463 in a way to facilitate charge translocation. Interaction essentially involves reorientations (rotation) of the side-chain rotamers of both the incoming arginine and F463 as the planar arginine guanidinium passes by the F463 ring. The hydrophobic properties of guanidinium above and below the molecular plane (Mason et al., 2003) facilitate its interaction with nonpolar groups, especially F463, of the hydrophobic plug and lower the energy barrier for arginine crossing. Aspartate D460, one turn above F463, stabilizes the translocated arginine in the external cleft of VSD.

In line with these observations and the notion that four arginine residues are involved in gating (Aggarwal and MacKinnon, 1996; Seoh et al., 1996), voltage sensors are thought to move in four sequential steps, each representing the passage of a different gating charge through the GCTC/hydrophobic plug. This view would simply represent an expansion of the model proposed by Aldrich and Sigworth groups (Zagotta et al., 1994a; Schoppa and Sigworth, 1998c) to include four sequential steps. Although some later studies questioned specific aspects of the idea of the GCTC (Pless et al., 2011; Lacroix and Bezanilla, 2011; Haddad and Blunck, 2011), the conceptual mechanism of this structure remains today in its essence, as confirmed by a number of structural and functional data accumulated in later years, that will be described below.

### Several studies strengthened the single-charge translocation’s view

Structural and functional data on Na and K channels accumulated in the following years strengthened the notion that during channel activation the S4 segment would visit several stable states when moving from the resting to the active position. Using MD methods to visualize the conformational changes of the Kv1.2 VSD during channel deactivation, Delemotte et al. (2011) found five stable states. In addition to the open (starting) state (α) and the resting (final) state (ε), the channel visited three stable intermediate states (β, γ, and δ), congruent with the S4 segment sequentially establishing and breaking ion pairs with nearby negatively charged residues, in a zipper-like fashion, as previously suggested by MacKinnon and coworkers (Tao et al., 2010). Similar conclusions were reached the following year by Elinder and coworkers who described a whole voltage-sensor gating cycle comprising one open and four closed states (Henrion et al., 2012). According to this study, the S4 segment would move for about 12 Å, from the active state (O) to the resting (C3) state, in this motion shifting three charges across the full membrane voltage drop. A deeper resting (closed) state C4 (the ε state of Delemotte et al. [2011]) could be reached with very large hyperpolarizations.

Elementary gating transitions occurring independently in each subunit and carrying a charge of ∼1e_0_ were reported by Tobias and coworkers using MD simulations of the isolated VSD from the KvAP channel upon hyperpolarizing the membrane from +100 to −100 mV and visualizing net gating charge displacements of ∼1e_0_ when arginine R6 moved from the external to the internal water cleft (Freites et al., 2012), and by Li et al. (2012) by studying the transitions of the VSD from Ci-VSP (a membrane-associated voltage-sensing lipid phosphatase enzyme from *Ciona intestinalis*) from the active and the resting states. From the crystal structures of the VSD from Ci-VSP in the active and resting state, they found that a displacement of S4 of only ∼5 Å (plus ∼60° rotation) was sufficient to switch the VSD between the two states, and this 5 Å “one-click” movement of S4 would bring arginine R4 to cross the hydrophobic plug, generating a net charge transfer or a current shot of ∼1e_0_ (Li et al., 2012).

Viewed with the state rate Markov models, these transitions and stable states would translate into an energy profile with five wells, separated by energy barriers that the voltage sensor must cross in succession while passing through the hydrophobic plug during activation. Viewed differently, the first of the four barriers would represent the energy that the first of the four gating charges is confronted with, the second barrier the energy for the second gating charge, and so on. Within this framework, each time a gating charge on S4 jumps over its energy barrier, i.e., crosses the hydrophobic plug (where the full membrane voltage is thought to fall), it should generate a current shot transporting a charge of 1.0e_0_.

### Limitations of gating current measurements

Gating current measurements have limitations at several levels. In general, they measure all the charges that move across the electric field (or part of it) following voltage changes, regardless of whether they are involved or not with the gating of the channel. This bias not only regards charges on the channel proteins under study but any membrane proteins with mobile charges or dipoles, which would then overestimate the effective gating charge. We must notice however that decades of investigation on gating current have shown a strong coherence, under varied experimental conditions, with several other approaches, indicating that, in spite of this obvious limitation, measurements of gating currents remain a reliable readout of voltage sensor movements and channel gating.

A second limitation is encountered at a deeper level of analysis of channel gating: gating currents cannot provide information on the individual movements of the voltage sensors in tetrameric or pseudo-tetrameric channels, as they average out the charges originating from their movement. Voltage clamp fluorometry has proven to be a valid approach to this regard and played a prominent role in clarifying this process, as we have partly addressed already. Chanda and Bezanilla used site-directed fluorescent labeling to follow the individual movements of the four S4 segments of the Na channel during activation in an effort to explain the slow component of the gating current in these channels, and found that it was likely due to the slow translocation of the S4 segment of domain IV during activation (cf., Fig. 5; Chanda and Bezanilla, 2002).

Another cluster of limitations of gating current measurement is the difficulty of appreciating slow charge movements with typical gating current protocols (that use pulse durations hardly longer than 500 ms). In the past, this inability to track the slow return of gating charges to their resting position upon repolarization has given rise to misinterpretations as charge immobilization or channel hysteresis. Voltage-clamp fluorometry had a prominent role in clarifying these processes, tracking the recovery from slow inactivation (Cowgill and Chanda, 2023) as well as fast inactivation (Savalli et al., 2007). The return of gating charges to their resting position after slow inactivation often manifests itself as hysteresis of the Q(V) curves—i.e., the observation that the Q(V) curve V_1/2_ is different depending on the “level” of inactivation. However, it has been postulated that hysteresis in Q(V) curves is not a genuine property of channel gating but a limitation of the experimental protocol (i.e., insufficiently long pulses) to resolve slow charge movements. Using improved voltage clamp fluorometry protocols in conjunction with gating current, Cowgill and Chanda demonstrated that the gating hysteresis arising from different initial conditions in Shaker K channel is eliminated with ultralong (18–25 s) test pulses, showing that the gating hysteresis is a kinetic feature rather than a true thermodynamic property of the channel (Cowgill and Chanda, 2023). Voltage-clamp fluorometry technique, in association with membrane conductance and gating current measurements, was used by Olcese and coworkers to investigate “charge immobilization” in BK channels (expressing also β_2_-inactivating subunit), using as reference the Shaker K channel, a classic model for N-type inactivation (Savalli et al., 2007). Unlike the Shaker channel where fluorometry data show the charge immobilization, as a consequence of N-type inactivation, they found no evidence of charge immobilization in BK channels (i.e., the β_2_-induced inactivation does not interfere with the gating charges returning to the resting position), suggesting an inactivating mechanism different from typical N-type inactivation (Savalli et al., 2007). In conclusion, like any other experimental technique, gating current measurements cannot give us a complete picture of voltage-gated channel gating. This can, however, be greatly expanded when the gating current study is done in conjunction with voltage-clamp fluorometry, or the emerging data on the atomic structures of the various conformations of the VSD.

## Gating currents and charge translocation at the microscopic level

### Gating currents are made of the ensemble current shots from S4 jumps

All the above information was not available in the late 1980s when Conti and Stühmer (1989) set out to uncover the atomic details of channel gating after the proposed sliding helix model. Their rationale was that the gating currents, mirroring the random jumps of these charged segments, were thought to produce shot-like current impulses. These infinitesimal shot currents were however impossible to see (to record) because of experimental limitations. Yet they ought to be “hidden” in the fluctuations (noise) of the macroscopic gating current, and, in principle, could be extracted with the analysis of noise applied to the fluctuations of the gating currents.

Conti and Stühmer applied the theory they developed to this end to the gating currents from macropatches of *Xenopus* oocytes expressing a large number of Na channels. The theory they used assumed two-state transitions for the charged elements (the voltage sensors). From the analysis of the gating currents noise, assumed to be produced by the single jumps or shot-like transitions of the S4 segments during activation, they determined the elementary apparent quantal charge (q_app_) associated with the shot-like transition to be 2.3e_0_. It was observed, however, that the significant filtering needed to contrast the background noise could be limiting for a reliable analysis of the fluctuations of the fast Na gating currents and be biasing the results (Conti and Stühmer, 1989). In any case, the shot charge obtained in this study was interpreted as an estimate of the amount of charge that moved in association with individual major transitions instrumental to open the channels. We should also recall that their theory was tailored for a two-state process. All this tells us that the shot noise expected then was not thought to be associated with the translocations of the individual charged residues on S4 across the membrane electric field, and thus not expected to be 1e_0_. A few years later, Bezanilla and coworkers applied noise analysis to the slower Shaker gating currents, paying special care to keep recording bandwidth as high as possible (Sigg et al., 1994). Notably, the shot charge they obtained was 2.4e_0_, essentially the same amount found for Na channels (also see Rodríguez et al., 1998).

### Crouzy and Sigworth’s comments on current shots from S4 jumps

Shortly after Conti and Stühmer (1989) had reported their noise analysis data on Na channels, Crouzy and Sigworth (1993) set out to extend Conti and Stühmer’s theory for a two-state process to discrete kinetic schemes of any complexity and investigate the effects of filtering. They found that the fast kinetics of the Na gating current combined with the modest time resolution could have prevented Conti and Stühmer from uncovering more complex kinetics. With regard to the shot current that Conti and Stühmer obtained from their noise analysis, and because of their limited time resolution, Crouzy and Sigworth further suggested that sequential gating charges could have crossed the gating pore in very rapid succession to become individually indistinguishable for the electrophysiology recording instruments, thus appearing as a single larger charge (multiple charge crossing; Crouzy and Sigworth, 1993). As a result, the value of 2.3e_0_, reported by Conti and Stühmer for the two-state model, could not report the real quantum charges crossing the pore, but the compound charges that pass through in rapid succession to result experimentally indistinguishable (because of the limited filter bandwidth).

Bezanilla and coworkers arrived at the same conclusion a few years later on observing an increasing value of q_app_ with increasing depolarization, from a minimum value of about 2.2e_0_ in the range of 0–20 mV to values close to 3.0e_0_ for depolarizations to +50 mV (Rodríguez et al., 1998; data reported below in Fig. 8 A). They suggested that the increasing values of q_app_ with increasing depolarization were due to the occurrence that limited time resolution of the recording system was showing more and more individual transitions merging as the depolarizing voltages increased and the time interval between consecutive charge passages through the hydrophobic plug decreased.

### A Brownian model of channel gating to address the issue at the atomic level

**Figure 6.**
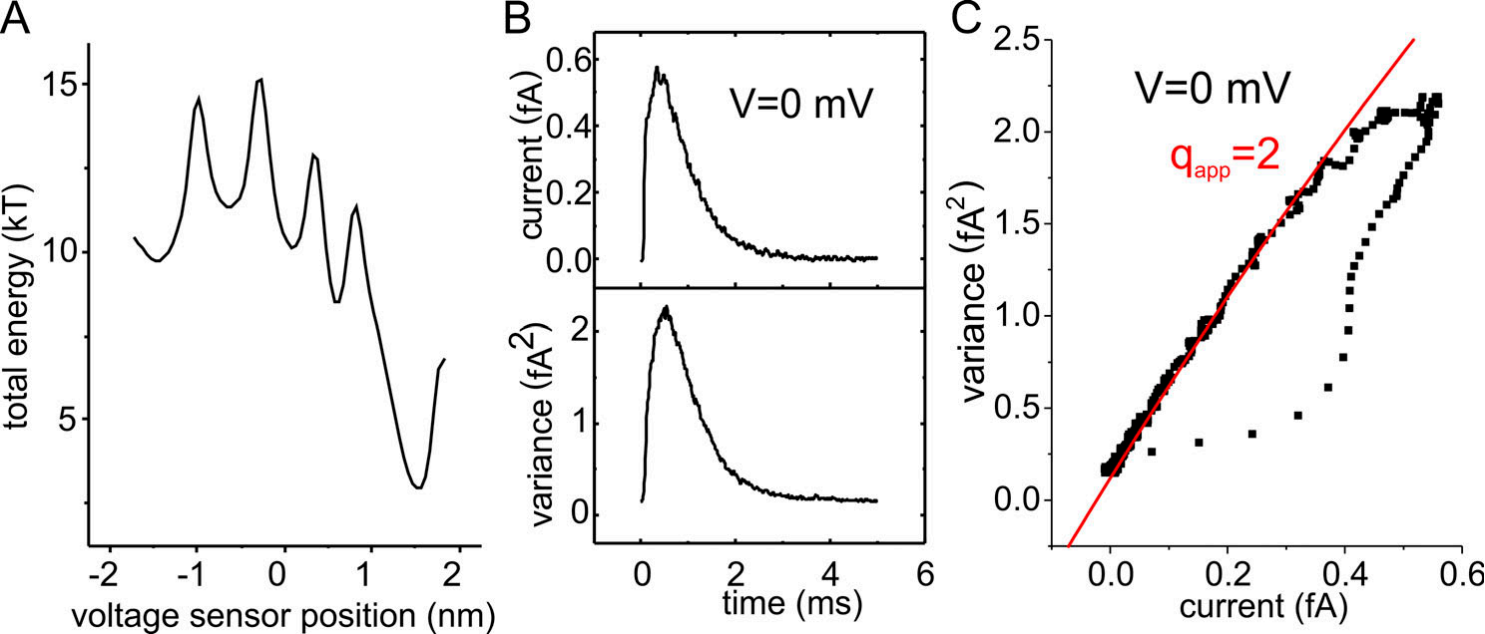
**Gating current fluctuations from our Brownian model. (A)** Total energy profile. **(B)** Plots of the mean current and variance. Microscopic currents in response to a depolarizing pulse to 0 mV were simulated 10,000 times, filtered with an eight-pole Bessel filter at a cutoff frequency of 8 kHz, and the resulting mean and variance assessed. **(C)** Plot of variance vs. mean current obtained from the energy profile of A. The solid line represents the best fit of the simulated data from the decaying part of the gating current using an equation derived from a single-step theoretical model of shot currents. The resulting apparent charge q_app_ is 2.1e_0_ (from Catacuzzeno et al., 2021a).

To readdress this issue of the elementary shot currents in channel gating and verify the proposition of Crouzy and Sigworth (1993) and Bezanilla and coworkers (Rodríguez et al., 1998), we used a Brownian model of channel gating developed to investigate at the atomic level the gating process of a K channel VSD (Catacuzzeno and Franciolini, 2019, 2022; Catacuzzeno et al., 2021b). The Brownian model was built on the structural details derived from crystallographic data of the Kv1.2 channel disclosed by MacKinnon’s group (Long et al., 2007) and on functional data on channel gating showing that during activation the voltage sensor visits five states (Delemotte et al., 2011; Henrion et al., 2012; Catterall, 2012) and translocates in its full motion three to four charges across the hydrophobic plug (this quantity is also congruent with the estimates of 12 to 14e_0_ transferred by one single Shaker channel upon full activation; Schoppa et al., 1992; Aggarwal and MacKinnon, 1996; Seoh et al., 1996). Accordingly, our Brownian model of voltage gating displays five states (five energy wells) in the energy profile and four energy barriers corresponding to the sequential crossing of the individual four gating charges on the voltage sensor through the hydrophobic plug (Fig. 6 A). The model was shown to well reproduce the major biophysical properties of the gating currents, as well as their fluctuations (noise; Fig. 6 B; Catacuzzeno and Franciolini, 2019; Catacuzzeno et al., 2021a).

Our initial tests showed, however, that the mean–variance relationship obtained with our Brownian model gave an apparent gating charge q_app_ of 2.1e_0_. This value was surprisingly close to 2.4e_0_ obtained experimentally on Shaker channels (Sigg et al., 1994), yet at odds with the now-established mechanistic view of voltage gating, envisioned as individual jumps of the four gating charges, one at a time, across the hydrophobic plug (the barriers in the Brownian model), and the q_app_ of 1.0e_0_ expected therein.

### A way out of the discrepancy between shot current and elementary charge

In trying to figure out the reason for the discrepancy, we considered the idea originally put forward by Crouzy and Sigworth (1993) to explain the noise analysis data of Conti and Stühmer (1989) on Na channels. That is, because of the limited filter bandwidth, sequential gating charges could cross the hydrophobic plug in very rapid succession to become individually indistinguishable from electrophysiology recording and appear as a single larger charge (multiple charge crossing). To this end, we analyzed a large number of simulations of a single-activating voltage sensor with our Brownian model to see if: (1) we could identify the elementary current shots supposedly originating when single gating charges on S4 pass individually through the hydrophobic plug; and (2) multiple charge crossings were indeed occurring during gating. Both elementary as well as multiple charge crossings were disclosed by the model.

**Figure 7.**
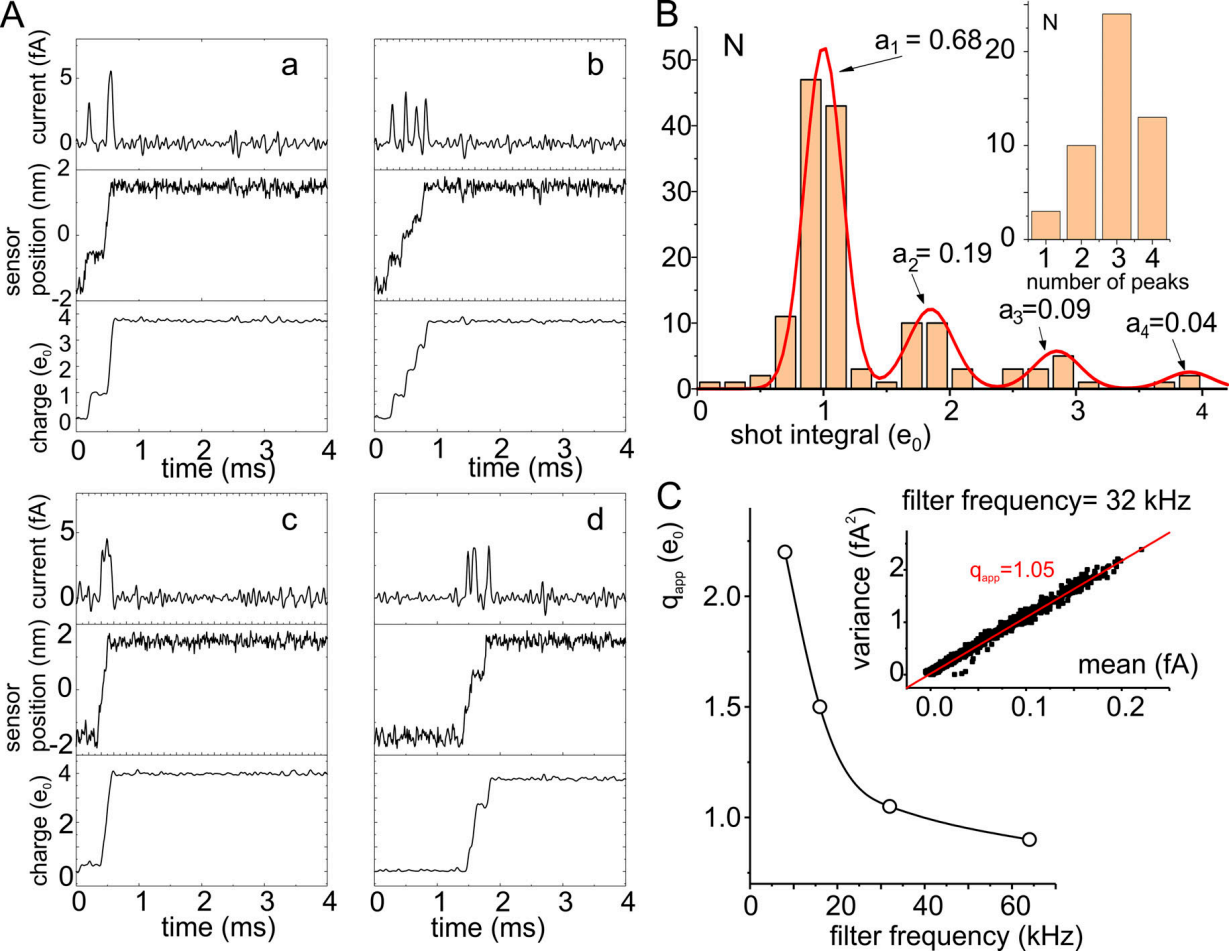
**Evidence for multicharge steps from a Brownian model of gating. (A)** Representative simulations upon pulsing to +10 mV (from a holding voltage of −110 mV), showing varying numbers of current peaks (a–d, top). For each simulation also shown are the voltage sensor movement and the charge transported. **(B)** Bar histogram of the number of events (N) carrying the indicated charge quantity (e_0_). Inset: Number of responses (N) with 1–4 peaks. **(C)** Dependence of q_app_ on the cutoff filter frequency. Data were obtained from Brownian simulated variance vs. mean current plots. The plot shown in the inset was obtained at a frequency of 32 kHz (from Catacuzzeno et al., 2021a).

We used our model to simulate the gating charge movement on applying a depolarizing pulse from −110 to +10 mV (the potential used by Bezanilla’s laboratory on Shaker, which gave a q_app_ of 2.3e_0_). The simulations showed high variability in the outcome of the elementary currents (shot events), in terms of number and size. Besides the simulations showing the expected four individual distinct peaks, each corresponding to crossing the hydrophobic plug by a single gating charge (Fig. 7 A a), we also obtained current responses with three, two, and also only one peak, as illustrated in Fig. 7 A, b–d, indicating that the passages of charges occurred in such a rapid succession that precluded to identify at various degree their individual passages. Frequency analysis of the 50 simulations done in this study (for a total of 147 peaks) showed that those with three peaks were the most commonly observed, followed by those with four, two, and one peak (Fig. 7 B).

The analysis also showed that in the simulations with four peaks, each peak was associated with the transfer of one unitary charge across the hydrophobic plug. In this case, the activation process arguably occurred in four distinct steps, with temporally separated passages of the four gating charges (Fig. 7 Aa). In the simulations with fewer peaks, suggestive of compound passages, we observed a strict reverse relation between the number of peaks and both the associated charge translocated and the voltage sensor movement.

These results are consistent with the notion that q_app_ larger than 1e_0_ indicates gating charges passing in rapid succession to make their individual jumps indistinguishable at the filter setting of the experiments. With the consequence that the more charges pass in rapid succession, i.e., “simultaneously” or “superimposed,” the higher the resulting q_app_ will be^3^. To confirm this view, we performed simulations at higher filter frequencies. As shown in Fig. 7 C, an increase in the filter cut-off frequency results in a decrease of q_app_ to values closer to unity. A representative simulated variance vs. mean current obtained with the Brownian model, using a Bessel filter at a cut-off frequency of 32 kHz, is shown in the inset.

### Dependence of the fluctuations (and multicharge crossings) on voltage

**Figure 8.**
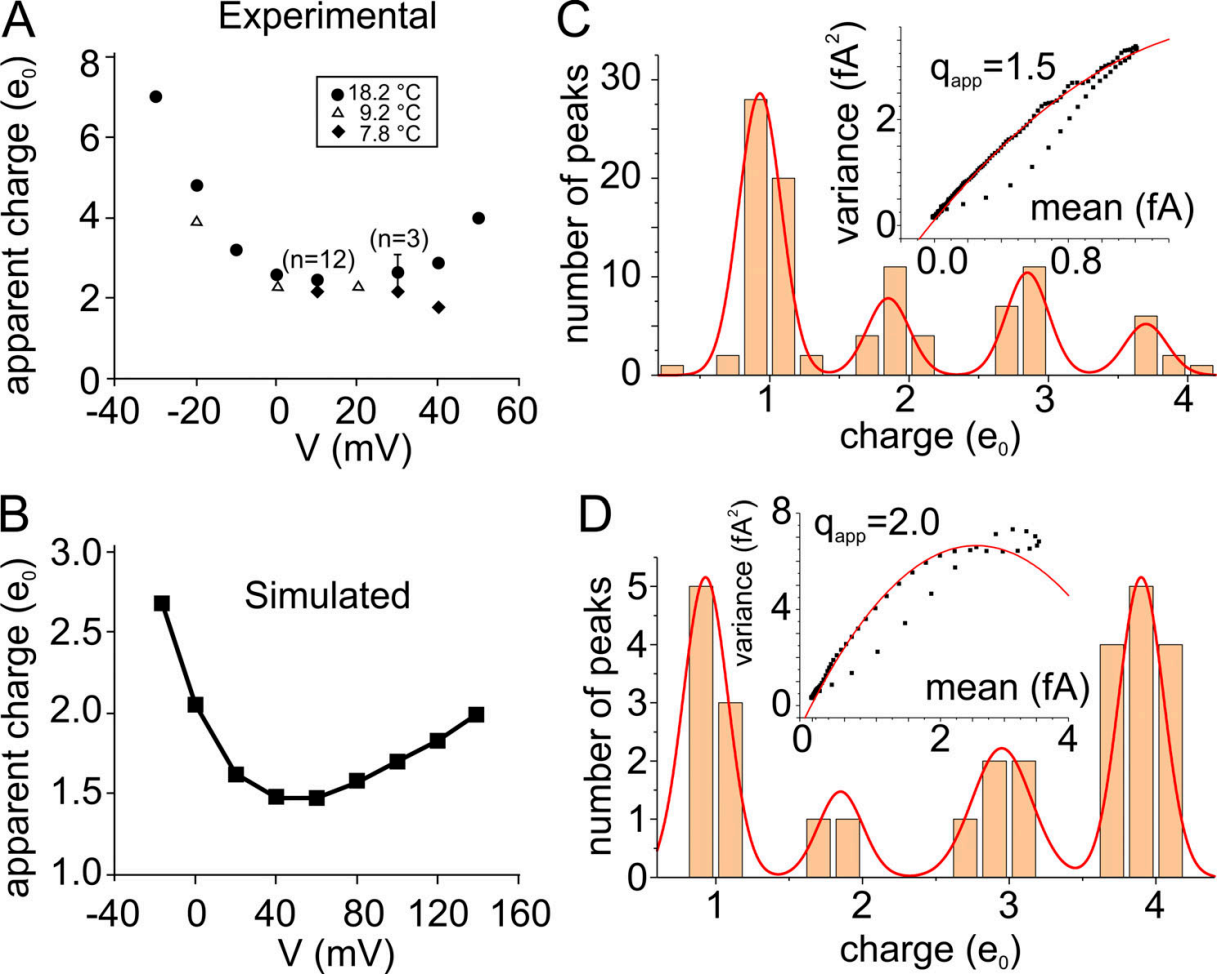
**Voltage-dependence of the apparent charge q**_**app**_**. (A)** Apparent charge (q_app_) vs. V relationship obtained experimentally by Rodríguez et al. (1998). **(B)** Apparent charge (q_app_) vs. V relationship simulated with the Brownian model by Catacuzzeno et al. (2021a). **(C and D)** Bar plots illustrating the number of peaks carrying 1, 2, 3, and 4 charges, respectively, at the applied voltages of +40 (top) and +140 mV (bottom), as assessed from the 40 simulations analyzed. Insets: Mean variance responses obtained at the same potential used in the corresponding histogram (from Catacuzzeno et al., 2021a).

Keeping up with their investigation on the fluctuation of the gating currents, Bezanilla and coworkers made another interesting observation: gating current fluctuations were strongly dependent on voltage, and the dependence was fairly peculiar, showing a marked U-shaped q_app_ vs. V relationship, with a minimum value of about 2.2e_0_ between 0 and +20 mV (Fig. 8 A; Rodríguez et al., 1998). When we tried to reproduce, with our Brownian model, the dependence of the gating current fluctuations on voltage, under conditions very similar to those used in Bezanilla’s laboratory, we also obtained a clear U-shaped relation, with a minimum q_app_ of about 1.5e_0_ at about +40 mV and a q_app_ of about 2.0e_0_ at +140 mV (Fig. 8 B). Reproducing fairly well the experimental findings of Bezanilla and coworkers, we possibly had the tool to really look into the mechanism generating the U-shaped relationship between q_app_ and voltage.

Keeping in mind the suggestion of Bezanilla’s group, whereby depolarization would increase the transition rates of the gating charges through the hydrophobic plug, and in turn, reduce the intercharge passage time and increase the chance (the fraction) of multicharge steps, and thus q_app_ (as also predicted by Eq. 14 in Catacuzzeno et al. [2021a]; see also (Catacuzzeno et al., 2020), we checked with our Brownian model the validity of this mechanism by assessing the rate of multiple charge crossing at +40 and +140 mV. The amplitude histogram of Fig. 8 C shows that at +40 mV peaks carrying one charge are the most frequent, although multicharge peaks are present. By contrast, at +140 mV peaks carrying one charge are greatly reduced in favor of those carrying four charges (Fig. 8 D). The strong increase in the fraction of multicharge peaks with depolarization, shown by these data, is arguably the (major) reason for the increase of q_app_ at more depolarized potentials observed experimentally by Bezanilla’s group (Fig. 8 A) and by our simulations (Fig. 8 B).

We have not yet addressed the other branch of the curve, the increasing q_app_ at higher hyperpolarizations. We notice though that the method to estimate q_app_ assumes irreversible (unidirectional) transitions of the voltage sensor (Conti and Stühmer, 1989; Crouzy and Sigworth, 1993), so the increasing values of q_app_ at potentials more negative than +40 mV can be thought to result from the increasing presence of backward transitions.

## Gating currents from other sources

We now briefly describe how gating current recording contributed to the comprehension of gating mechanisms of voltage-dependent channels other than classic Na and K presented above, as the Ca channels where gating currents were first recorded, the large-conductance and Ca^2+^-activated K (BK) channel where voltage gating integrates with Ca^2+^ gating, and the hyperpolarization-activated cyclic nucleotide (HCN) channel, where gating displays a reversed polarity. We will then describe the gating currents from voltage-gated structures lacking the canonical ion pore domain, as the proton channels (Hv1), and finally a voltage-gated protein outside the realm of ion channels, as the enzyme voltage-sensing phosphatase (VSP).

### Gating currents from Ca channels

Ca channels carry a special meaning here for being the ion channels where gating currents were first recorded (Schneider and Chandler, 1973). Schneider and Chandler succeeded in this effort by blocking pharmacologically the principal ion conductances of the frog skeletal muscle fiber chosen for their study, and applying a subtraction procedure of digitized records taken at different voltages to remove the large linear capacitative current that would cover the small charge displacement current (Schneider and Chandler, 1973).

**Figure 9.**
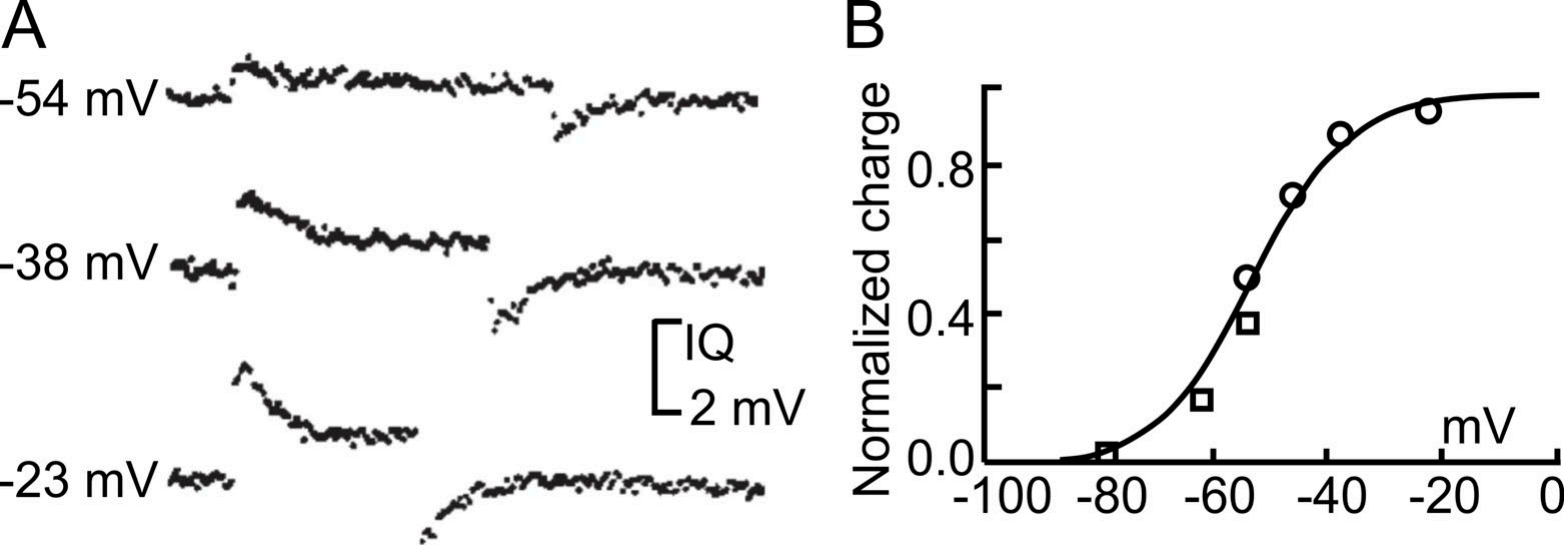
**First recordings of gating charge currents from ion channels. (A)** Gating currents obtained by subtracting from the currents recorded at the indicated potentials the currents obtained with the same voltage pulses applied at a membrane potential range where channels do not activate (negative to resting potential). Pulse duration was shortened as depolarization increased. **(B)** Voltage dependence of the ON gating charge is described by a Boltzmann function, as expected from the classic gating model. Circles represent measurements from the experiment in A; squares are measurements at earlier times from a similar set of experimental traces (modified from Schneider and Chandler, 1973).

The recordings obtained with this protocol disclosed the presence of asymmetric, transient currents whose amplitude and kinetics increased with depolarization (Fig. 9 A). Schneider and Chandler further observed that the ON charge translocated in response to varying depolarizing steps was well described by a Boltzmann function (Fig. 9 B), consistent with the classic notion of a homogeneous population of gating particles moving back and forth between two position in the membrane in response to voltage changes. These results and the observation that the voltage dependence of the recorded asymmetric currents overlapped the voltage range for muscle contraction made Schneider and Chandler believe that these currents were generated by the movement of the voltage sensors associated with the excitation–contraction coupling of the skeletal muscle, that is, the dihydropyridine receptors or L-type Ca channels present on the T-tubule membranes (Rios and Brum, 1987).

In a series of studies from the late 1980s on frog muscle fibers—the actual source of Schneider and Chandler’s gating current—Rìos, Stefani, Brum, and coworkers tested the effects of low extracellular Ca^2+^ and varied holding potentials on Ca^2+^ release from the sarcoplasmic reticulum (SR) and on intramembrane charge movement (Brum and Rios, 1987; Brum et al., 1988b; Brum et al., 1988a). As for the second aspect, more relevant to this retrospective, they found that depolarized holdings or conditioning prepulses markedly affected transmembrane charge movement: i.e., reduced the charge that moves during depolarizing pulses (i.e., from −100 to 0 mV), termed “charge 1” in muscle fibers (Adrian and Almers, 1976), while increased the charge moved by hyperpolarizing pulses in the very negative range (i.e., from −100 to −180 mV), termed “charge 2.” They also quantified the amount of charge 2 in muscle fibers inactivated by long depolarization, and found that it was essentially the same as that of charge 1 that was possible to record from fibers kept well polarized.

That work (Bezanilla et al., 1982; Fernández et al., 1982; Brum and Rios, 1987; Brum et al., 1988b; Brum et al., 1988a) established the 2-mode (Available—Inactivated), 4-state model (Closed, Open, Open inactivated, Closed inactivated) shown below (from Brum et al., 1988a; modified). According to the model, channels can be in one of two clusters of functionally distinct states or modes, here separated by the horizontal dotted line: the Available mode, represented by the Resting and Active states, and the Inactivated mode, represented by the two inactivated states illustrated, both nonfunctional since unable to couple excitation to contraction. In the model, only the horizontal transitions are voltage dependent and associated with charge (and voltage sensors) movement. *Cis* and *trans* indexes indicate in fact the position of the voltage sensors: on the intracellular side of the membrane electric field (down) and on the extracellular side (up), respectively. Vertical transitions are instead voltage independent.

**Scheme 3.**
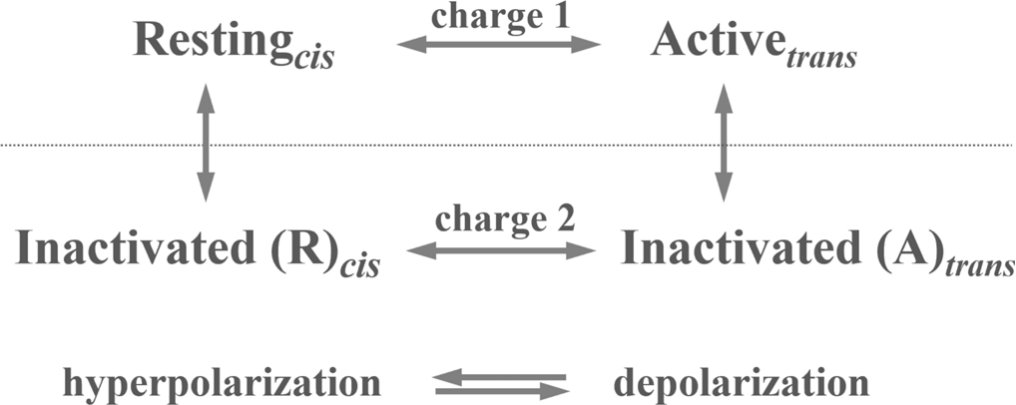
**Rìos and coworkers’ 2-mode, 4-state model of charge interconversion in muscle Ca channels.** See Gating currents from Ca channels for full description (from Brum et al., 1988a).

According to the model, strong depolarizations from negative holdings drive channels from the Resting_*cis*_ (closed) to the Active_*trans*_ (open) state while translocating charge 1 (cf. Scheme 3). If the depolarization is maintained, the Active_*trans*_ (open) channel enters the Inactivated (A)_*trans*_ state. From this Inactivated (A)_*trans*_ state, channels can be driven into a second inactivated state, the Inactivated (R)_*cis*_ state, if a strong hyperpolarization is applied. This last transition is associated with the translocation of charge 2 to the down position.

Those studies showed that charge 1 and charge 2 present differences but also commonalities. They differed markedly in the voltage needed to move them across the electric field (their Q(V) V_1/2_ were well separated on the voltage axis). They were instead similar in the maximum amount of charge carried, and the temporality with which they seemed to appear/disappear (increase/decrease; Brum and Rios, 1987; Brum et al., 1988b; Brum et al., 1988a). Based on these observations, these authors proposed that charge 1 and charge 2 were the same charges originating from the same structures (the voltage sensors), only dwelling in different modes, and, as result, displaying distinct properties that depended on the mode (Available or Inactivated) they were in. The implication of this view was that charge 1 and charge 2 could interconvert into one another. Notably, in the interconversion process, channel inactivation stabilized the associated charge while recovery from inactivation removed this stabilization. From the Inactivated (A)_*trans*_ state (where virtually all channels end up upon long depolarization) with charges (charge 2) stabilized in the up position, channels could be moved into the second inactivated state, Inactivated (R)_*cis*_, and the charges in the down position, but strong negative potentials were needed. This last observation, more than anything, showed that charges did not get “immobilized” with inactivation, as was also being proposed at the time (Hadley and Lederer, 1989; Hadley and Lederer, 1991). In a later study on mammalian (mouse) muscle fibers, Brum, Rìos, and coworkers would show even more convincingly interconversion of charge 1 and charge 2, in full agreement with the 4-state model illustrated above, by showing the conservation of the sum between the two types of charge and same time course of their switching mode upon varying the duration of the depolarization (Ferreira Gregorio et al., 2017).

To study these aspects in more detail, Ferreira and coworkers measured the gating currents from expressed cardiac L-type Cav1.2 channels (which share most biophysical properties with Cav1.1 of skeletal muscle) and related them to the progression of voltage-dependent inactivation (using Ba^2+^ as charge carrier; Ferreira et al., 2003). They first confirmed the V_1/2_ shift of the Q(V) curve to more negative voltages upon inactivation, as found in Cav1.1 channel, with the voltage shift increasing with progression of inactivation, congruent with charge interconversion. They also found that V_1/2_ shift as function of inactivation (i.e., of depolarization length) was described by a double-exponential function, which they attributed to the simultaneous development of two distinct processes: charge immobilization and charge interconversion, both associated with channel inactivation. Based on converging evidence gathered from their studies, they suggested that the fast process (τ ∼0.5 s) was associated with N-type fast inactivation and charge immobilization, while the slower process (τ ∼4 s) was associated with C-type slow inactivation and charge interconversion (Ferreira et al., 2003).

The 2-mode, 4-state model presented above can satisfactorily describe both inactivation processes due to the addition of the Inactivated (R) state to the classic 3-state model (Resting_*cis*_ ↔ Active_*trans*_ ↔ Inactivated (A)_*trans*_, to stay with the above nomenclature) used to explain the main features of N-type fast inactivation and charge immobilization of many typical voltage-gated channels. This Inactivated (R)_*cis*_ state is inactivated-closed, with the voltage sensor in the *cis* position (down), and can be reached following strong hyperpolarizations, which also brings along the translocation of charge 2. Similar phenomena have been described in many voltage-gated channels as well as Ci-VSP, and have been associated with a “relaxed” (inactive) state of the VSD induced by prolonged depolarization (Bezanilla, 2018). Variants of this model have been used to describe the inactivation processes in several voltage-gated channels (Marks and Jones, 1992; Kuo and Bean, 1994; Olcese et al., 1997; Roux et al., 1998).

Years later, Olcese and coworkers used voltage-clamp fluorometry to follow the movement of the four S4 voltage sensors in an effort to identify their individual roles in Ca channel (human Cav1.2) gating (Pantazis et al., 2014). We recall that Ca channels share the pseudo-tetrameric structure of the Na channel, displaying four chained domains (I–IV), each formed by six transmembrane segments (S1–S6), of which S1–S4 form the VSD. Based on the voltage- and time-dependent movements of the four voltage sensors, as derived from voltage-clamp fluorometry experiments, they concluded that VSD-II and VSD-III were chiefly responsible of Ca channel opening, although the involvement of VSD-I, which also displayed quite fast activation kinetics, could not be excluded. Surely excluded from participating in channel activation was instead VSD-IV because of its much slower activation kinetics (Pantazis et al., 2014).

Flucher and coworkers used instead domain swapping between the VSD-I and VSD-IV on Cav1.1 channels to examine in more detail the role of VSD-IV that structural models had suggested to be involved in channel activation (Tuluc et al., 2016). They found that both VSDs are involved in channel activation, VSD-I in controlling the speed of channel opening, VSD-IV in establishing the voltage threshold, but neither VSD was found to be involved in channel inactivation (Tuluc et al., 2016). These results were later confirmed by the same group who reexamined the question using a combination of techniques (MD modeling, site-directed mutagenesis, and electrophysiology; Fernández-Quintero et al., 2021). Flucher and coworkers noticed the contrast of their results with the fluorometric study of Olcese’s group on Cav1.2 channel, where only VSD-II and VSD-III appeared involved with channel activation (Pantazis et al., 2014). As a tentative explanation, they highlighted the different channel type studied (Cav1.1 vs. Cav1.2), and the unique functional role of Cav1.1 (in the activation of ryanodine receptors RyR1) that may warrant a different gating mechanism (Tuluc et al., 2016). A few years later, Olcese and coworkers investigated the individual biophysical properties for each of the four voltage sensors of the human skeletal muscle Cav1.1 channel, using electrophysiology, fluorescence measurements and computational modeling. They found that the four VSDs exhibit different properties, congruent with the following specific functions: VSD-I governs Cav1.1 channel activation while VSD-II and VSD-III (and possibly VSD-IV) are involved in excitation–contraction coupling of muscle fibers and sarcoplasmic reticulum Ca^2+^ release (Savalli et al., 2021).

Gating current measurements were also used to gain insights on the mechanism by which Ca channel accessory subunits modulate coupling between the VSD and the pore domain. Stefani and coworkers expressed rabbit cardiac Ca channel α_1_ subunit in *Xenopus* oocytes, assessed the steady-state voltage dependence of charge movement and pore opening and found that the half-activation potential for charge movement was about 35 mV more negative than for pore opening (Neely et al., 1993). When the α_1_ subunit was coexpressed with the cardiac Ca channel β subunit this difference was drastically reduced, without affecting the Q(V) curve. Thus, intramolecular coupling between the voltage sensor and the channel pore opening can be facilitated by regulatory subunits (Neely et al., 1993).

A few years later, Josephson and Varadi (1996) tested the effects of the human cardiac Ca channel β subunit on the L-type Ca channels expressing α_1_ + α_2_ on the gating charge movements (gating currents) and the pore opening. While they found that the Ca current density increased fourfold and the gating currents nearly fivefold in the presence of the β subunit, which may be due to a different expression efficiency (Lory et al., 1993), the most important result was that neither the ion current nor the gating current voltage dependence was affected by the α_1_ subunit, and as consequence the relationship between the Q(V) and G(V) curve (Josephson and Varadi, 1996). The reason for the discrepancy with earlier experiments remains to be clarified.

### Gating currents from BK channels

**Figure 10.**
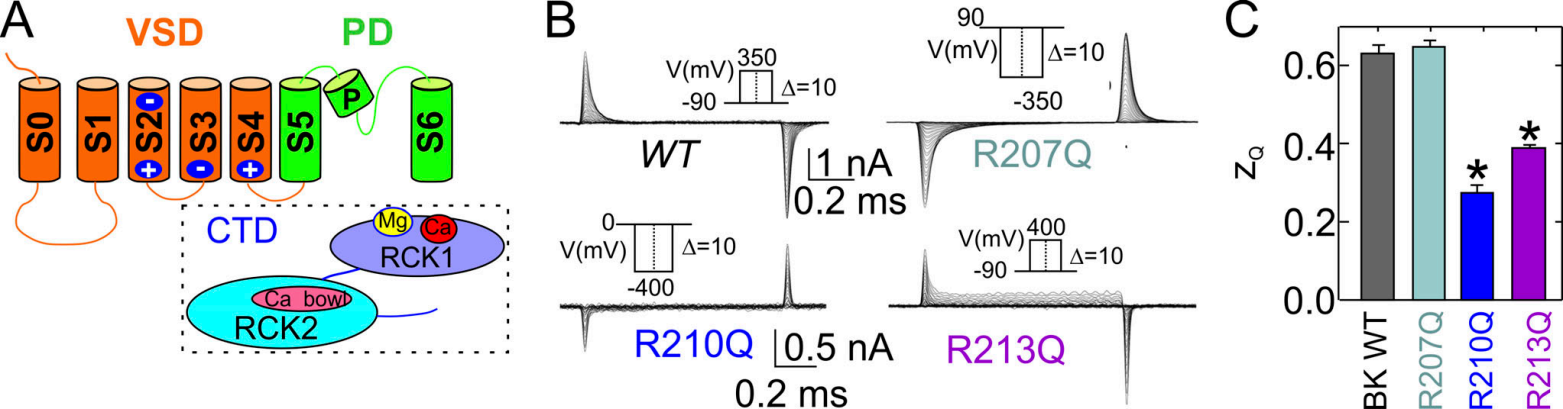
**Gating currents from BK channels. (A)** Cartoon of the voltage and Ca^2+^-gated BK channel highlighting the VSD, the pore domain (PD), and the Ca^2+^-sensing domain (CTD) (from Yang et al., 2015). **(B)** Representative gating current records of WT and neutralization mutants (indicated) in the S4 helix of the human BK channel. **(C)** Gating charges displacement (z_Q_) for BK WT and the mutants shown in B (modified from Carrasquel-Ursulaez et al., 2022).

Gating currents have been profitably used to investigate the voltage dependence of the large-conductance and Ca^2+^-activated K (BK, also known as Slo1 or KCa1.1) channels, widely present in virtually all cells, and activated allosterically by membrane depolarization and intracellular Ca^2+^. BK channels display the tetrameric architecture typical of the 6TM voltage-gated superfamily, with four α subunits, each containing the transmembrane segments S1–S4 that form the channel’s VSD and the segments S5 and S6 concurring in making up the pore domain. A further N-terminal S0 segment, important for interaction with auxiliary subunits, is also present (Liu et al., 2008). The BK channel contains in addition a bulky cytosolic domain carrying the high-affinity sites for Ca^2+^ binding, that serve for Ca^2+^ modulation of channel opening (Fig. 10 A; Jiang et al., 2002; Xia et al., 2002; Sweet and Cox, 2009).

Focusing on the voltage dependence, the BK channel was found to display three arginine residues on the S4 segment (R207, R210, and R213 in the human BK), that is, only one arginine less than those found to be relevant in Shaker channel gating (Aggarwal and MacKinnon, 1996; Seoh et al., 1996). Yet, despite the still-significant number of putative gating charges present on S4, it was calculated that only ∼2.4e_0_ per channel moved upon full channel activation (Stefani et al., 1997; Horrigan and Aldrich, 1999, 2002). This figure, which is about fivefold smaller than in the Shaker channel (∼13e_0_; Aggarwal and MacKinnon, 1996; Seoh et al., 1996), appears difficult to be attributed to the different number of gating charges present in the BK channel S4 (three vs. four). Either not all the arginine residues on S4 are involved in gating or different gating mechanisms are operating in this channel.

Latorre and coworkers investigated the role of individual charged residues on S4 of BK channel (hSlo) using the limiting slope method, and found that only neutralizations of R210 and R213 reduced the gating charges (Díaz et al., 1998). Horrigan’s laboratory later recognized, however, that this method in BK channels underestimates the total gating charge (Ma et al., 2006). They therefore used the limiting slope as an initial indicator of changes in gating charge or electromechanical (EM) coupling, but ultimately estimated the gating charge by fitting data over a wide range of voltages and Ca^2+^ concentrations with the Horrigan–Aldrich gating scheme (Horrigan and Aldrich, 2002). Their results showed that unlike classic voltage-gated K channels where the gating charge is concentrated on the S4 segments, BK channels show diffuse distribution of gating charges, with only one (voltage-sensing residue) on S4 segment (R213), two charged residues on S2 segment (D153 and R167) and one on S3 segment (D186; Ma et al., 2006), and all of them participating in channel gating (Savalli et al., 2006, 2007; Pantazis et al., 2010).

To understand how this charge-decentralized VSD functions, more specifically to assess if cooperative functional interaction between the canonic S4 voltage sensor and S2 (which carries the highest charge) exists, Olcese and coworkers have optically followed the rearrangements of the two variously neutralization-mutated BK VSD transmembrane helices during activation (Pantazis et al., 2010). Their results show that S2 and S4 segments are both sensitive to voltage and, more importantly, they interact functionally. That is, neutralization of the gating charge in one segment reduces the effective valence (the voltage dependence) of the other (Pantazis et al., 2010). Allosteric models of the experimental findings suggest two concurring mechanisms: in addition to the canonic physical interaction between the VSD and the pore gate a second mechanism has been proposed that consists in the dynamic (activation-dependent) rearrangement of the membrane electric field. In other words, activation of one charged segment could modify the aqueous clefts, focusing the field over a shorter dielectric distance, with the result that the other charged segment that moves over the same physical length would cross a larger fraction of the membrane voltage drop, which would result in a higher measured gating charge (Pantazis et al., 2010).

The results of Ma et al. (2006) indicated that, of the three arginine residues on S4, only R213 contributed to the gating charge, and its neutralization reduced by only 0.3e_0_ the charge translocated within each single S4 (i.e., 1.2e_0_ per channel; to compare with a total of ∼13e_0_ per channel of Shaker, or ∼1e_0_ for each gating charge neutralized; Aggarwal and MacKinnon, 1996; Seoh et al., 1996). In light of the weak coupling between the S4 movement and the pore opening displayed by BK channels (Horrigan and Aldrich, 1999; Cui and Aldrich, 2000), the extremely low charge translocated upon channel activation reported above could be the result of the limiting slope shortage to reliably estimate the gating charge movement, as the method measures in fact the voltage dependence of channel opening. With the consequence that for weakly coupled channels the two parameters (values) may not be strictly linked.

To bypass these potential experimental limitations, Latorre and coworkers estimated the charge displacement directly from the gating currents of mutant channels where each arginine on the S4 of BK channels was in turn neutralized (Carrasquel-Ursulaez et al., 2022). The analysis of the gating currents showed that only the neutralization of residues R210 and R213 drastically modified the voltage dependence of charge translocation (z_Q_; Fig. 10, B and C). Some additional features of BK channel gating currents are worth mentioning. First, they are ∼100 times faster than those of Shaker-like Kv channels (and much faster than BK channel opening), which is the reason why they can be characterized in detail despite a fivefold lower gating charge per channel. Second, they seem consistent with a single-step VSD activation (i.e., single exponential kinetics and single Boltzmann Q(V), if measured while channels are closed). Both features could be understood in terms of the mechanism of charge movement proposed in Carrasquel-Ursulaez et al. (2022). Especially important, MD simulations^4^ showed that BK channels display a gating mechanism different from classic Kv channels where the gating charges cross entirely the hydrophobic plug (the GCTC) and reach the opposite water-filled cleft of VSD. By contrast, in BK channels, which do not have an equivalent to the GCTC, the gating charges R210 and R213 are only partly displaced, upon full voltage activation, within the thin hydrophobic wall (the counterpart of the hydrophobic plug in K channels) that separates the internal and external water-filled clefts of the VSD (Carrasquel-Ursulaez et al., 2022). This means that they only cross a fraction of the whole membrane voltage drop, and this is why the BK channels show weak voltage dependence.

BK channels are activated allosterically by voltage and internal Ca^2+^ (Barrett et al., 1982; Horrigan and Aldrich, 2002; Latorre et al., 2017). To understand the role of Ca^2+^ in the coupling between charge movement and pore opening, Ligia Toro and coworkers investigated the effect of Ca^2+^ on gating currents. They found that gating currents can be recorded upon depolarization also in virtual absence of Ca^2+^ (<5 nM in inside-out patches), indicating that channel activation can be induced in a Ca^2+^-independent manner (Stefani et al., 1997). Increasing cytosolic Ca^2+^ they also found that the Q(V) curves were shifted to more negative voltages, indicating that Ca^2+^ facilitates the translocation of the voltage sensors, or switches the channel into a gating mode where it requires less voltage to open (Stefani et al., 1997). These results show that BK channels can operate in a Ca^2+^-independent and in a Ca^2+^-dependent mode, and that micromolar Ca^2+^ favors the switch to the Ca^2+^-modulated mode where the equilibrium is shifted toward the open states (Stefani et al., 1997). These data were later confirmed by Olcese and coworkers who used voltage-clamp fluorometry and UV photorelease of intracellular caged Ca^2+^ to optically follow the VSD activation triggered by Ca^2+^ binding to the gating ring (i.e., a raise of cytoplasmic Ca^2+^ levels shifted the voltage dependence of both VSD activation and channel opening; Savalli et al., 2012). They made, however, a crucial observation: impairing the Ca^2+^ binding to RCK2 domain, but not to RCK1, abolished the effect of [Ca^2+^]_i_ increase on the VSD rearrangements, revealing that the two high affinity Ca^2+^ sensors of the human BK channel, RCK1 and RCK2, are not functionally equivalent (Savalli et al., 2012).

### Gating currents from HCN channels

We now address the HCN (hyperpolarization-activated cyclic nucleotide gated) channel, prominently found in pacemaker cells of the heart and self-firing neurons of the brain where it concurs to their cyclic excitation (McCormick and Pape, 1990; DiFrancesco, 1993). When the currents associated with this channel were first recorded (from the heart sino-atrial cells), they were unexpectedly found to be activated by hyperpolarization, which granted them the name of “funny currents,” *I*_*f*_, or “hyperpolarization-activated currents,” *I*_*h*_ (Brown et al., 1979). These currents are in addition directly modulated by cAMP that binds to the C-terminus cytosolic domain of the channel, which explains the sympathetic modulation of the heart rate (Robinson and Siegelbaum, 2003).

Although the reverse voltage activation of the *I*_*f*_ might have suggested a voltage sensor carrying negative charges, the HCN channel cloning (Seifert et al., 1999; Monteggia et al., 2000) showed that it was instead structurally similar to the other members of the 6TM voltage-gated ion channel superfamily, displaying six putative transmembrane segments (S1–S6), with the S4 segment carrying multiple positively charged residues interposed by two uncharged residues (Seifert et al., 1999; Kaupp and Seifert, 2001).

Unfortunately, gating currents could not be recorded from the small cells of the heart nodes where these currents were first recorded, nor from the other sources studied later. The gating mechanism of HCN channel had thus to be addressed with different strategies. Larsson and coworkers used substituted-cysteine accessibility methods to assess whether the S4 segment, the putative voltage sensor, worked in the canonical way, moving through the VSD during activation (Männikkö et al., 2002). To this end, they assessed the thiol reagent MTSET accessibility of several cysteine mutant HCN (spHCN) in S4, at varying voltages, and showed that S4 moves upward in response to a depolarization, as typical voltage-gated K channels do.

To gain more in-depth kinetic information on the voltage sensor movement and its dependence on voltage, the same laboratory exploited fluorescence signals as reporters of the S4 movement (obtained by introducing a cysteine in the lower part of S4, in place of arginine R332), in conjunction with ion and gating current measurements (Bruening-Wright et al., 2007). Their results indicated that S4 in spHCN channels makes two sequential but distinct movements in response to depolarization: a fast motion that translocates considerable charge but does not open the channel, and a second slower motion carrying less charge and associated with channel opening (Bruening-Wright and Larsson, 2007). They further observed that the ion current traces superimposed with the fluorescence traces when these were raised to a power of two, suggesting that channel opening is dependent on the prior occurrence of two distinct events, in other words, the channel can open after only two S4 segments have moved. As HCN channels are gated by voltage in a reverse way from typical Kv channels, they must have a different coupling mechanism between VSD and PD (Männikkö et al., 2002).

### Gating currents from Hv1 channels

The voltage-gated proton (Hv1) channel, expressed on CNS microglia and in some immune cells (Capasso, 2014; Wu, 2014), controls cellular pH by passing proton currents out of the cell upon depolarization (Cherny et al., 2003; DeCoursey et al., 2016; De La Rosa and Ramsey, 2018). In 2006, *C. intestinalis,* mouse, and human *Hv1* genes were cloned, and the related Hv1 channels inferred to have a peculiar structure made only of the voltage sensing domain, that is, four transmembrane segments (S1–S4), with S4, putatively the channel voltage sensor, carrying three highly conserved arginine residues (R201 [R1], R204 [R2], and R207 [R3] in mouse Hv1), and S1, S2, and S3, carrying several negatively charged residues that interact with and stabilize the S4 gating charges while passing by (Ramsey et al., 2006; Sasaki et al., 2006; Gonzales et al., 2009).

Native Hv1 channels assemble as homodimers (Tombola et al., 2008; Lee et al., 2008; Koch et al., 2008), although each monomer has its own permeation pathway and can function independently, when separated (Tombola et al., 2008; Koch et al., 2008). Despite lacking the typical pore domain for ion permeation, the Hv1 channel can mediate the proton current by making a water pathway for H^+^ passage across the hydrophobic septum of the VSD. In the dimeric form, the Hv1 channel gates cooperatively: both monomers must translocate their voltage sensor before either proton pathway can conduct (Gonzalez et al., 2010; Musset et al., 2010; Tombola et al., 2010; Smith and Decoursey, 2013). Using the limiting slope method on Ci-Hv1 channels expressed in oocytes, Larsson and coworkers estimated the effective gating charge displaced by the monomeric and dimeric form to be 2.7e_0_ and 5.9e_0_, respectively (Gonzalez et al., 2013).

Because of the initial hardship in recording the gating currents from Hv1 channels, the first clues on channel gating were obtained from cysteine accessibility methods, voltage-clamp fluorometry and classic electrophysiology. These studies essentially showed that the S4 segment moves upward in response to depolarization and all the three charges (arginine residues) on S4 contribute to the channel’s voltage dependence, although none are indispensable (Gonzalez et al., 2010; Gonzalez et al., 2013).

**Figure 11.**
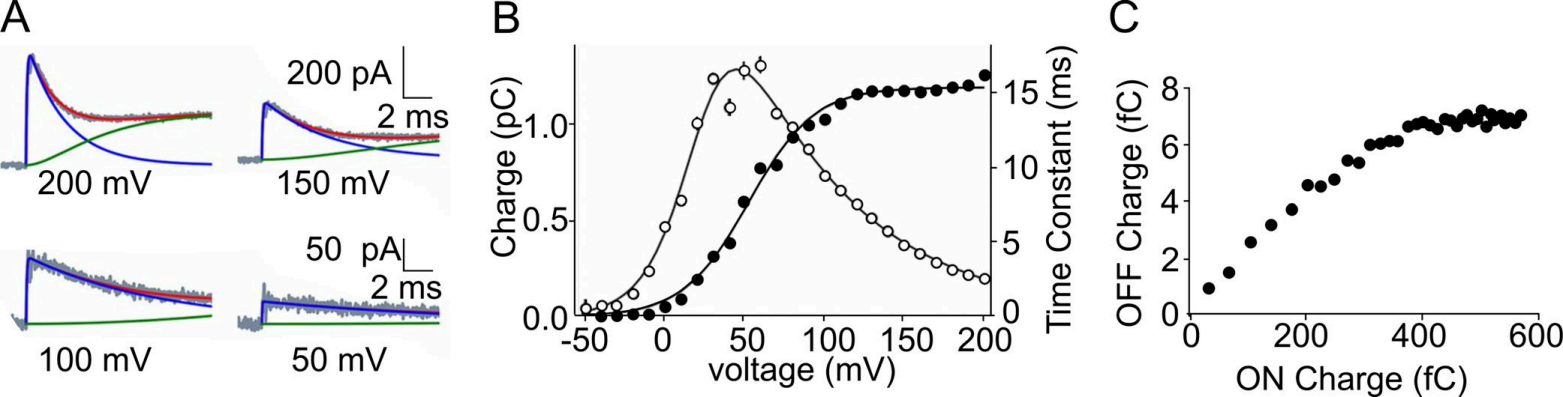
**Gating currents from voltage-gated Hv1 channel. (A)** Experimental currents from monomeric Hv1 mutant N264R (black traces) in response to varying depolarizing pulses (level indicated) from a holding potential of −70 mV. The fitting procedures isolated the ON-gating current (blue traces) from the ion current (green traces). **(B)** Charge displacement as a function of voltage, Q(V) (filled circles). The experimental data were fitted by a Boltzmann function (solid line) with V_1/2_ = 52.8 mV and zδ = 1.2. Open circles represent the time constants of the gating current decays at the given depolarization and were fitted with a two-state model (solid line). For fitting parameters, see the original work (Carmona et al., 2018). **(C)** Plot comparing the OFF- and ON-gating charge obtained with pulses of different durations (from Carmona et al., 2018).

In 2018, two groups succeeded in reliably recording the gating currents from Hv1 channels (De La Rosa and Ramsey, 2018; Carmona et al., 2018). The gating currents reported by Carmona et al. (2018) showed kinetics congruent with the activation of the proton current, and saturation with large depolarizations, as expected for genuine gating currents (Fig. 11 A). The complex time course of the ON-gating current suggested that the voltage sensor experiences multiple conformational transitions upon activation, although one of them seems to carry most of the charge (Fig. 11 B). The ON- and OFF-gating currents isolated analytically were instead unexpectedly unbalanced, even for short pulses, with the OFF component making only a few percent of the ON current (Fig. 11 C). Although this observation may remind of some form of extreme charge immobilization, in this case it was suggested to be due to the arginine side chain at 264, introduced to minimize the proton current (Hong et al., 2014; Carmona et al., 2018). The analysis of the gating currents done by De La Rosa and Ramsey also showed that the voltage sensor moves in several steps and adopts many different conformations upon activation, and neutralization of arginine residues on S4 reduces in parallel the amount of the gating charge translocated (De La Rosa and Ramsey, 2018). The gating currents also showed biophysical features that suggest thermodynamic coupling of voltage sensor activation and pore opening.

The data from these two studies are most valuable as they have provided important insights for a first thorough characterization of the gating mechanism of Hv1 channels. We must bear in mind, however, that both studies, to minimize the ion H^+^ current, used Hv1 channels carrying a mutation (N264R) that introduced an additional charge on the S4 voltage sensor. All this is to say that the data reported by the two studies should not be overemphasized.

In 2020, Tobias and coworkers obtained structural models of the open and closed states of the Hv1 channel by applying MD simulations at varying potentials, using as putative model for the closed state the crystallographic structure of the chimeric construct of Takeshita et al. (2014) based on mouse Hv1 (mHv1cc; Geragotelis et al., 2020). The hyperpolarized configuration shows the S4 voltage sensor fully pushed inwards, with the arginine side chains pointing toward the cytoplasm. Strong depolarizations induced an immediate displacement of the first (topmost) gating charge (R205) toward the extracellular side, above the hydrophobic plug, defined by F150, without any appreciable movement of the S4 segment and the other two gating charges, R208 and R211, that remained on the intracellular side, below the hydrophobic plug. The second displacement event induced by depolarization brought the S4 segment upward by ∼8 Å, and R208 to jump past F150, on to extracellular side (R205 was instead pushed further up, at the interface of the membrane). R211 remained instead at the level of F150, and never reached the extracellular side. The total charge displaced by depolarization was estimated to be ∼2.7e_0_ (Geragotelis et al., 2020), consistent with previous experimental data (Gonzalez et al., 2010; Fujiwara et al., 2012; De La Rosa and Ramsey, 2018).

### Gating currents from Ci-VSP

**Figure 12.**
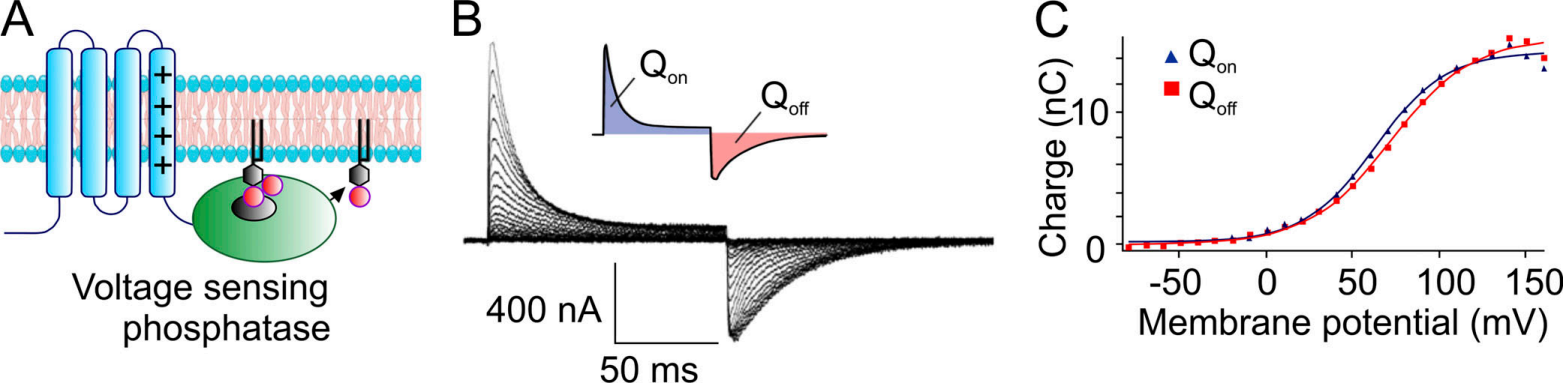
**Gating currents from voltage-gated phosphatase (Ci-VSP). (A)** Cartoon of the voltage-gated lipid phosphatase enzyme highlighting the VSD module (blue) and the enzymatic moiety (green). **(B)** Gating currents at varying depolarizations from voltage-gated lipid phosphatase. Inset shows the equivalence of the ON- and OFF-gating currents. **(C)** Charge translocation associated with the ON and the OFF component, as a function of voltage (from Okamura et al., 2018).

We finally describe the contribution of gating currents in elucidating the gating of the VSP, a protein family widely present across all phyla, with the property of removing phosphate groups from various substrates. In 2006, the membrane-associated lipid phosphatase enzyme from *C. intestinalis*—a PTEN-related phosphoinositide phosphatase (Ci-VSP)—was reported to be regulated directly by membrane potential (Murata et al., 2005). Ci-VSP was found to have a voltage sensing domain totally similar to the VSD of voltage-gated channels, formed by four transmembrane segments (S1–S4), the fourth of which contained four arginine residues as putative gating charges (R223, R226, R229, and R232), arranged in the typical every-third-position and assumed to function as a voltage sensor (Murata et al., 2005; Villalba-Galea, 2012). The VSD of Ci-VSP (Ci-VSD) also had in its middle section the typical cluster of hydrophobic side chains (made of I126, F161, and I190) that would make an effective dielectric barrier between the intra- and extracellular water-filled clefts, as the hydrophobic plug or the GCTC in Kv channels (Campos et al., 2007; Tao et al., 2010). There is however a major difference in the overall architecture compared with classic voltage-gated channels: the Ci-VSP is a monomeric protein with only one VSD (Fig. 12 A).

The VSD of Ci-VSP is easy to separate from the catalytic moiety and investigate in isolation (Kohout et al., 2008). When subjected to depolarizing pulses, the VSD produces gating currents similar in shape to voltage-gated channels (Fig. 12 B), but with a much slower time course and a Q(V) curve heavily moved rightward (V_1/2_ ∼ +60 mV; Fig. 12 C; Murata et al., 2005). This heavily shifted Q(V) curve, far beyond 0 mV, makes the voltage sensor assume the non-active (resting) conformation during protein crystallization (as the method zeroes the membrane potential). This occurrence becomes even more interesting in light of the fact that the Q(V) curve can be moved leftward by >100 mV by neutralizing arginine R217 (the outermost charge on S4), and has a voltage sensor stably in the active conformation at 0 mV (Kohout et al., 2008).

The elucidation of the Ci-VSD crystal structures in both the active and the resting conformations allowed to estimate an upward movement of S4 by ∼5 Å upon activation, and an associated net charge translocation of ∼1e_0_ across transmembrane electric field (the hydrophobic septum). Notably, this value is very similar to earlier estimates from gating current studies (Murata et al., 2005; Villalba-Galea, 2012). The crystal structures showed that this one-click switch, i.e., an ∼5 Å translocation of S4, specifically corresponds to the gating charge R226 (R2) jump across the hydrophobic septum (Li et al., 2014). That the translocation of only one single gating charge across the hydrophobic septum switches the system on and off explains the weak voltage dependence of Ci-VSP. The voltage sensor of Ci-VSD could, however, undergo a larger translocation than indicated by the resting and activated crystal structures (cf. Li et al., 2014), for example, make a two-click switch that would translocate the S4 voltage sensor by about 10 Å and double the charges that cross the septum, and in addition be more congruent with metal-ion bridge data reported by Lindahl, Elinder, and coworkers (Henrion et al., 2012). In this case, the conformation following the one-click switch would only be an intermediate state of Ci-VSD. It is interesting to recall here that fusing the Ci-VSD to the voltage-independent viral K channel Kcv results in a perfectly functional chimera (KvSynth1), that is, in a voltage-gated K channel that can be activated by only one-click of its VSD, as in the activation of Ci-VSP (Arrigoni et al., 2013).

Most recently, Perozo and coworkers have carried out MD simulations to estimate the S4 displacement and the significant conformations the (isolated) Ci-VSD encounters during a full gating cycle (Shen et al., 2022). In addition to the activated “Up” and resting “Down” states identified earlier from crystallographic studies (Li et al., 2014), the simulations disclosed two new VSD states: a deeper resting state (“Down-minus”) and an extended activated (“Up-plus”) state (Shen et al., 2022). Their results, further supported by cysteine accessibility and metal-ion bridges experiments, suggest that voltage activation of Ci-VSD comprises four conformational states and involves the sequential crossing of the three arginine residues on S4 through the membrane electric field, which results in the translocation of a total of ∼3e_0_ charges (Shen et al., 2022).

## Coupling voltage sensor and pore domain

In voltage-gated channels, the conformational rearrangement of the voltage sensor induced by a depolarization (the upward movement) is transferred to the pore domain that rearranges to open the ion conduction pathway. In these channels, the pore domain is formed by the juxtaposition of the four S5–S6 segments interlaced through the P-loops, which converge to form the selectivity filter and control ion selection. Ion permeation is instead controlled by an intracellular gate formed by the C-terminal ends of the S6 segments that come together at the cytoplasmatic side of the channel to form the bundle crossing (Holmgren et al., 1998). At resting, negative potentials, the bundle crossing is collapsed, forming a hydrophobic seal impermeant to ions (Doyle et al., 1998; Del Camino and Yellen, 2001; Hackos et al., 2002; Xu et al., 2019). Upon depolarization, the pore (the bundle crossing) opens as a result of the upward translocation of the voltage sensor that is covalently linked to the pore via S4–S5 linkers, suggesting that these structures serve to convert the electrical energy released by membrane depolarization into mechanical work to open the channel pore, possibly acting as mechanical levers (Labro et al., 2005; Long et al., 2005b).

### EM coupling in classic voltage-gated channels

The first evidence of the role of the S4–S5 linker in EM coupling of classic voltage-gated ion channels came from the analysis of substitutions of the highly conserved penta-leucine repeat present in the region that connects and partly overlaps the terminal portion of S4 and S5, which was shown to severely affect Shaker channel gating (McCormack et al., 1991). This notion was strongly validated by later observations that the voltage-independent bacterial K channel KcsA, with subunits made of only the two segments involved in ion permeation (S5 and S6), could be made voltage-dependent by fusing it with the Shaker VSD, as long as the S4–S5 linker was included (Lu et al., 2001).

Some doubts remained, however, as the S4–S5 linkers were directly connected with the S5 segments, but it was the S6 segments that controlled the opening and closing of the pore. These doubts seemed to be dispelled by a follow-up study by the same group showing that two complementary sequences in Shaker, one located in the distal part of the S4–S5 linker and the other at the cytoplasmic end of S6 segments that form the bundle crossing, were both needed for coupling (Lu et al., 2002). Other mutagenesis studies that targeted these domains also showed the crucial role of several residues present there in EM coupling (Ficker et al., 1998; Ding and Horn, 2003; Ferrer et al., 2006; Soler-Llavina et al., 2006; Labro et al., 2008; Muroi and Chanda, 2009; Haddad and Blunck, 2011). The crystal structures of Kv channels were perfectly congruent with these conclusions as they also showed a major non-covalent interacting zone between the S4–S5 linker and the cytoplasmic end of S6 (Long et al., 2005b; Long et al., 2007).

These crystal structures not only showed a direct interaction between the S4–S5 linker and S6 terminal, but they also revealed that the voltage sensors and the pore domains did not form tight protein–protein interactions. These observations helped establish the canonical mechanism of EM coupling for classic Kv channels (subgroup Kv1–Kv7) and for structurally homologous Na and Ca channels, with the S4–S5 linker as the central element, and its interaction with terminal S6 segments as the basic mechanism that link the S4 voltage sensor activation to pore opening. In addition to the idea that the S4–S5 linkers wrap around the pore to hold the channel closed when the VSDs are in the resting position (Pathak et al., 2007; Long et al., 2007; Wisedchaisri et al., 2019), it should be noted that the S4–S5 linker in the activated VSD also contacts S6 (Long et al., 2007), and these interactions have been suggested to contribute to EM coupling by helping to “pull the channel open” (see review by Blunck and Batulan, 2012).

The key role of S4–S5 linker seemed, however, strongly challenged when Pardo and coworkers, using a split channel strategy, reported that the voltage-gated *ether-a-go-go* EAG1 (Kv10.1) channel, a member of the KCNH family that also includes ERG (Kv11) and ELK (Kv12) channels, could still be gated about normally by voltage, regardless of the removal of the S4–S5 linker (Lörinczi et al., 2015), indicating that this structure was not required to provide voltage dependence to these channels. The role assigned to the S4–S5 linker in EM coupling would instead be played by the terminal portion of the S4 segment or the initial remaining part of the S4–S5 loop which becomes the effective structures to transfer in a non-covalent manner the conformational changes experienced by the S4 voltage sensor to the S6 bundle crossing to open and close the pore. This interpretation was consistent with previous mutagenesis studies showing that in another member of the KCNH family, the hERG channels (Kv11.1), these two structures interact significantly (Sanguinetti and Tristani-Firouzi, 2006; Ferrer et al., 2006), and with another study on hERG that used the same split channel strategy and showed that the removal of large portions of the S4–S5 linker would barely affect voltage gating (de la Peña et al., 2018).

Cryo-EM structures of the EAG channel (the channel studied by Pardo’s group), that followed shortly, showed that they do not have the domain-swapped architecture displayed by classic Kv channels and typical voltage-gated Na and Ca channels (Whicher and MacKinnon, 2016). In the non-domain-swapped structure of the EAG channel, the VSD of one subunit faces and interacts with the S5–S6 segments of the same subunit. The S4–S5 linker is in addition much shorter compared with domain-swapped channels, suggesting that in these channels, given the close proximity between the terminal part of S4 and the S6 segment forming the pore, EM coupling could be attained by direct interaction between these two domains, with no requirement for the S4–S5 linker (Whicher and MacKinnon, 2016). The role of the S4–S5 linker remains instead in its full formulation for domain-swapped voltage-gated channels, where the S4 voltage sensor and the pore S6 segment of the same subunit are much farther apart, making direct contact impossible. The following year, the cryo-EM structure of the open human EAG-related K channel hERG showed that it shared the same non-domain-swapped architecture and associated features with the EAG channel, and supposedly the same EM coupling mechanism (Wang and MacKinnon, 2017).

In the following years, new observations indicated that the canonical pathway in EM coupling of domain-swapped channels might not be the only one operating in channel gating. Using a new interaction-energy analysis, Chanda and coworkers found robust interactions also between the S4 and S5 segments, besides those already observed between the S4–S5 linker and S6 (Fernandez-Marino et al., 2018). Using the Shaker channel and a tandem dimers approach, Carvalho-de-Souza and Bezanilla (2019) showed that interactions between S4 and S5 were also important in controlling another aspect of the overall functional state of the pore, namely the C-type inactivation that affects the selectivity filter and in turn the channel P_open_. The study also showed that this non-canonical coupling operates over short-range distances (within van der Waals forces). In a later investigation, the same laboratory identified the putative residues connecting the S4 voltage sensor to the selectivity filter as the structural elements of non-canonical coupling (Bassetto et al., 2021). These studies opened the way to the notion that EM coupling could occur through two separate pathways: a canonical pathway involving the S4–S5 linker and a non-canonical pathway based on direct interaction between the S4 and S5 segments. In most EM coupling studies, the translocation of the S4 voltage sensor from resting to the activated state was implicitly assumed to occur through a one-step transition, although it was well-known that it needed to pass through several states. The question then was whether the molecular domains involved in EM coupling, as well as the underlying mechanism, were S4-state invariant.

To address this point, Tarek, Cui, and coworkers (Hou et al., 2020) exploited the peculiar feature of the domain-swapped Kv7.1 channel of having two clearly distinct open states associated with two different positions of the voltage sensor (Zaydman et al., 2014; Barro-Soria et al., 2014). Using electrophysiologic approaches and MD modeling, they showed that the mechanism/interactions between the significant elements of EM coupling differed for the two distinct translocations of the voltage sensor that bring Kv7.1 into the two distinct open states. Namely, during the first S4 transition from rest to the intermediate activated state, which brings the pore to its first open state, the (“hand-like”) C-terminal portion of the S4–S5 linker interacts with the pore domain of the same subunit, that is, following the canonical gating interaction. By contrast, the second coupled transition that moves the S4 voltage sensor further outward to its fully activated state and the pore into its second open state involves the interaction of a different structure of the VSD, namely the “elbow-like” hinge placed between S4 and S4–S5 linker, with the pore domain of the adjacent subunit (Hou et al., 2020). Notably, these non-canonical interactions not only involve a different domain of the VSD but they occur with the pore of a different subunit. Ensemble covariance analyses, used to identify co-evolved protein residues (Rivoire et al., 2016), indicated that the two-stage “hand-and-elbow” gating mechanism may apply to other domain-swapped Kv channels (Hou et al., 2020). In fact, the non-canonical pathway illustrated above has several commonalities with non-canonical interactions recently reported to contribute to the EM coupling in Shaker channel (Fernández-Mariño et al., 2018; Carvalho-de-Souza and Bezanilla, 2019).

### EM coupling in BK channels

We saw earlier that BK channels integrate voltage and Ca^2+^ gating to fully exert their physiological functions. As a member of the voltage-gated K channel family, the BK channel is made of four identical α subunits (Slo1), with a voltage sensing (S1–S4) and a pore (S5–S6) domain. They differ, however, from the other members of the family for having an additional S0 transmembrane segment (which interacts with the β subunit) and a cytosolic terminal (Ca^2+^ binding) domain (CTD) in continuity with the pore-forming S6 segment. The CTD is made of two RCK domains (regulators of conductance for K^+^) per subunit (RCK1 and RCK2) that carry the Ca^2+^ binding sites (Hou et al., 2009).

Unlike classic Kv channels, BK channels display non-domain-swapped architecture and a very short S4–S5 linker. Interactions between S4 and S5 (i.e., between VSD and PD) are in addition far larger, suggesting a major role for these segments in EM coupling, possibly exerted through non-canonical pathways (Tao et al., 2017). Besides interacting with the pore domain, the VSD of BK channels also makes extensive contacts with the CTD, which in turn contacts extensively the pore domain. This arrangement could potentially mediate a novel form of indirect voltage-dependent coupling that could also integrate Ca^2+^ modulation. Congruent with this notion, the removal of the CTD or specific mutations (i.e., L390P) at the VSD/CTD interface degrades markedly voltage-dependent coupling (Zhang et al., 2017; Geng et al., 2020).

Sun and Horrigan performed site-directed mutagenesis of several residues on the S4–S5 linker, S6 tail, C-linker, and part of the cytoplasmic Ca^2+^ domain with alanine as a substitute (alanine scan) to assess their role in voltage-dependent coupling (Sun and Horrigan, 2022). Their results showed that mutations in the S4–S5 linker have no significant consequences on the voltage dependence of channel gating (opening). The cytoplasmic portions of S4, S5, and S6 were instead found to establish interactions suggestive of a non-canonical coupling that would transfer the activation-bound conformational change of S4 to S5, which in turn stabilizes the cytoplasmic ends of S6 in the unfolded state (pore open; Sun and Horrigan, 2022). In any event, in their modeling, Sun and Horrigan assumed that coupling is weak. Using patch-clamp fluorometry associated with mutagenesis or auxiliary subunits manipulation (Miranda et al., 2018), or by examining the effects of internal Ca^2+^ on BK channel gating currents (Carrasquel-Ursulaez et al., 2022), it was found that the coupling between Ca^2+^ binding and voltage sensing (i.e., between CTD and VSD) is strong, with the result that voltage-dependent gating in BK channels is strongly modulated by internal Ca^2+^.

Sun and Horrigan’s data disclosed a second, indirect pathway that connects the S4 voltage sensor to the cytoplasmic Ca^2+^ domain, the C-linker, and eventually the S6 segment. They also showed that the C-linker, which is covalently interposed between the S6 segment and the cytoplasmic Ca^2+^ domain, plays its coupling role exclusively via extended non-covalent interactions with its bordering domains. This indirect pathway has been suggested to help stabilize the pore in the closed state when the S4 voltage sensors are in the resting state (Sun and Horrigan, 2022). This shows that the indirect pathway includes the cytoplasmic Ca^2+^ domain (which exerts its own Ca^2+^ control over pore opening), and as such establishes that the two pathways are not independent.

Summarizing, the voltage-dependent coupling in BK channels is thought to proceed through two main pathways (direct and indirect) that differ in their structural elements, biophysical mechanisms, and channel gating role. The direct pathway within the VSD is at work when the S4 voltage sensors are in the activated state and serves to stabilize the pore open. The indirect pathway, through Ca^2+^ and CTD, stabilizes instead the closed conformation of S6 helices at rest. This pathway, which also integrates the cytoplasmic Ca^2+^ domain and its modulation on pore opening, is controlled by both voltage and cytoplasmic Ca^2+^ (Sun and Horrigan, 2022). The actions of the two pathways are, however, non-additive, so that full activation of either one or both simultaneously produce the same effect (Sun and Horrigan, 2022), somehow rectifying the classic proposition of voltage and Ca^2+^ effects being essentially independent and additive in BK channel activation (Cui and Aldrich, 2000; Horrigan and Aldrich, 2002).

### EM coupling in HCN channels

Unlike classic voltage-gated channels, the hyperpolarization-activated HCN channels activate, as the name says, on hyperpolarization, although their S4 voltage sensors display positive charges and move upward in response to depolarization (Männikkö et al., 2002). These observations suggested that the inverted voltage dependence of HCN channels was due to a reversed EM coupling. The cryo-EM structure of HCN1 in the resting state, reported by Lee and MacKinnon (2017), showed that, like the related EAG and hERG channels, the HCN channel displays a non-domain-swapped structure, including a particularly long S4 segment that would allow unique interactions with the pore domain.

Comparing this structure with the cryo-EM structure of the HCN1 in an activated state, reported shortly afterward (Lee and MacKinnon, 2019), and with data from MD simulations (Kasimova et al., 2019), fluorescence resonance energy transfer, and Rosetta modeling (Dai et al., 2019) revealed peculiar conformations and movements of the S4 voltage sensors. First, with the voltage sensor in the activated state (at depolarized voltages), both the cryo-EM structure and MD simulations showed the pore in the closed state. Second, the S4 voltage sensors move downward considerably (∼10 Å) in response to hyperpolarization. Third, on moving downward, the lower portions of the S4 segments bend markedly with respect to their original axis (and their upper portions) and assume a position parallel to the membrane, reminiscent of the S4–S5 linker orientation at rest. Fourth, channel opening in response to robust hyperpolarization (i.e., to −100 mV) was associated with the translocation of two gating charges across the membrane electric field. It thus appears that the conformational changes of HCN VSD in response to voltage follow those of typical voltage-gated channels, with the S4 voltage sensors moving upward in response to depolarization and downward upon hyperpolarization. By contrast, pore opening has inverted voltage dependence, occurring upon hyperpolarization, when the S4 voltage sensors have reached a downward position. This could be explained by a reverse coupling between the voltage sensor and the pore (Lee and MacKinnon, 2017; Dai et al., 2019; Kasimova et al., 2019).

A number of studies showed, however, that specific mutations at the cytoplasmic end of the S4 and S5 segments could reverse the voltage dependence of gating in these channels, making them open upon depolarization, or disclose the full potential of the channel to open the pore in either voltage direction (Flynn and Zagotta, 2018; Kasimova et al., 2019; Ramentol et al., 2020). Some of the data from these studies appeared in addition incongruent with the reverse coupling hypothesis (Kasimova et al., 2019). In any event, altogether they showed that non-covalent interactions between S4 and S5, through residues that are highly conserved in HCN channels, were important in their EM coupling (Flynn and Zagotta, 2018; Kasimova et al., 2019; Ramentol et al., 2020).

Larsson and coworkers replaced several conserved residues individually and used voltage clamp fluorometry and ion current recordings to assess simultaneously the movement of the S4 voltage sensor and the functional state of the pore (Wu et al., 2021). They focused on two conserved residues of the sea urchin HCN channel, E356 and N370, that the cryo-EM hHCN1 structure places between the lower parts of S4 and S5. Mutation of either one with alanine, which arguably prevents, because of its small size and chemistry, the interaction (the formation of hydrogen bonds) between S4 and S5, shifted rightward the voltage dependence of S4 movement by about 70 mV. In other words, the mutations destabilize the S4 in the upward position or, to put it differently, the interaction between E356 and N370 (i.e., between S4 and S5) stabilizes S4 upward (Wu et al., 2021).

Mechanistically informative were also the following two observations: (1) while the voltage dependence curve of S4 movement in E356A mutant channels was shifted to more positive voltages (by 70 mV), the voltage dependence curve of channel opening (measured as ion current) was shifted to more negative voltages; and (2) mutation E356A disclosed a second distinctive fluorescence component that appeared in the voltage range −60 to −160 mV, indicative that the S4 voltage sensor would move in two steps, each with specific properties. It was further found that stepping to −50 mV (from 0 mV), where the fluorescent signal was nearly maximal, and so was the gating charge translocation as assessed through gating currents, the channel did not open. This suggested that the movement of the major fraction of the gating charge, occurring with the first translocation step of S4, was unable to open the pore (Wu et al., 2021).

As for the second fluorescence component, i.e., the second translocation step of S4 that brings its lower portion to move out from the original axis, it was found to occur within the same voltage range of the pore opening and to have virtually the same time course, suggesting that it was this second translocation step of S4 to open the pore. Cysteine accessibility experiments and MD simulations suggested that this second conformational change of S4 segments induces the formation of internal water clefts between the lower parts of the S4 segments and the pore domain that would make room for the lateral shift of the intracellular ends of S6, that is, open the pore (Wu et al., 2021). It may be of interest to mention the gating case of the KAT1 channel, a K-selective inward rectifier cloned from the higher plant *Arabidopsis* (Hedrich, 2012). Like HCN channels, KAT1 channels expressed in *Xenopus* oocytes are activated by hyperpolarization, although the voltage sensors undergo an upward movement during activation (Latorre et al., 2003). Based on the cryo-EM structure of KAT1, which also disclosed its non-domain-swapped architecture, Peroso and coworkers (Clark et al., 2020) proposed a direct mechanism of electromechanical coupling that contrasts in several respects with allosteric mechanisms proposed for hyperpolarization-activated HCN channels (Altomare et al., 2001; Chen et al., 2007). Namely, they suggested that under depolarized conditions, the voltage sensors interact with the closed pore domain directly, primarily with S4 and S5 overlaying the C-linker of the adjacent subunits. The hyperpolarization-driven inward movement of the S4 voltage sensors induces a conformational change of the C-linkers that ultimately opens the cytoplasmic activation gate (bundle crossing).

## Voltage sensor channelopathies

The VSD is a common target for mutations that cause inherited disorders or diseases. In light of the focus of the present retrospective on gating currents, here we will give a few examples of pathological mutations targeting specifically the gating charges on the VSD of voltage-gated channels, which understandably are the most frequently found (Matthews et al., 2009).^5^ A substantial number of these mutations were found to cause hypokalaemic periodic paralysis (HypoPP), a pathological phenotype characterized by episodic severe muscle weakness and paralysis episodes associated with marked hypokalemia and unusually depolarized muscle membranes (during the attacks) that preclude the generation of action potentials (Venance et al., 2006). Often HypoPP arises from mutations targeting arginine residues in the S4 voltage sensor of either Na (Nav1.4) or Ca (Cav1.1) channel, suggesting a common pathological mechanism (Struyk and Cannon, 2007).

A first hint toward its comprehension came with the observation that the typical depolarized condition recorded from biopsies of muscle fibers from HypoPP patients was insensitive to TTX or nitrendipine (blockers of Na and Ca channels, respectively; Ruff, 1999), indicating that the depolarization did not result from dysfunction of (i.e., leak through) the canonical permeation pore of these channels. The explicit finding was the detection of the “gating pore currents” (or ω currents) through the voltage sensor module of HypoPP mutant skeletal muscle Nav1.4 channels (Sokolov et al., 2007; Struyk and Cannon, 2007). These currents were initially observed in Shaker K channels on replacing the topmost S4 arginine residues with smaller amino acids. This substitution would open a conductance pathway for inward unselective passage of cations at hyperpolarized voltages (including resting) that was not associated with the central, canonical permeation pore but with the formation of a water pathway within the voltage-sensing domain, and give rise to the gating pore currents (Tombola et al., 2005). Although these currents are normally very small (a few percent of the central pore current), they were found to be sufficient to induce a significant depolarization of Nav1.4 mutant-expressing cells (Sokolov et al., 2007) and make them unable to generate action potentials, the hallmark of HypoPP (Sokolov et al., 2007; Struyk and Cannon, 2007).

The proposition that the gating pore currents cause the HypoPP was consolidated when later observations consistently showed that several other Nav1.4 HypoPP mutants, tested by functional expression, all produced gating pore currents at negative potentials (i.e., in resting cells; Kubota et al., 2020). Also Cav1.1 HypoPP mutants (all targeting arginine residues on S4) were shown, initially indirectly (Wu et al., 2012; Fuster et al., 2017) and then more stringently (Kubota et al., 2020), to produce gating pore currents at negative potentials.

Another form of periodic paralysis that occurs under normokalaemic serum level, NormoPP, was found to be determined by mutations that specifically neutralize the R3 gating charge on Nav1.4 channel (Vicart et al., 2004). Because of the lower position of R3 on S4, these mutations, unlike those causing HypoPP, generate gating pore currents only when the voltage sensor is in the activated position, that is, at highly depolarized voltages (Sokolov et al., 2008). As these voltages are seen by the cell only for brief time intervals, say during the overshoot of the action potential, these small gating pore currents of NormoPP mutant channels were initially thought of little consequence as to inducing the excitability dysfunctions seen in NormoPP disorders. Catterall and coworkers however observed that the gating pore current in Nav1.4 NormoPP mutant channels remains active also in slow-inactivated Nav1.4 channels. As a result, the gating pore current will increase concurrently with the building up of slow inactivation that occurs during trains of action potentials needed to evoke the tetanic contraction, and become sufficiently large to cause pathological dysfunctions (Sokolov et al., 2008).

More recently, the same laboratory has obtained high-resolution structures of the bacterial Na channel NavAb incorporating mutation R2G or R3G, analogous to those found to cause HypoPP and NormoPP, respectively (Jiang et al., 2018). Molecular modeling showed that both mutations were found to form aqueous pathways with different features across the VSD hydrophobic plug, whose continuity, and thus ion permeation, was differently controlled by voltage: HypoPP mutant R2G displayed gating pore currents under resting conditions, whereas NormoPP mutant R3G showed it at positive potentials (Jiang et al., 2018).

The generation of the gating pore currents has been taken as an explanation of the dysfunctions (mainly ventricular arrhythmias) caused by another group of mutations targeting the S4 voltage sensor of Nav1.5, the main Na channel expressed in the heart. Three mutations, targeting the gating charges on the S4 of Nav1.5 VSD I (R219H, R222Q, and R225W; R1, R2, and R3, respectively), and a fourth mutation targeting R3 on S4 of Nav1.5 VSD II (R814W), all found in unrelated patients suffering from ventricular arrhythmias and dilated cardiomyopathy (Gosselin-Badaroudine et al., 2012b; Moreau et al., 2015b; Jiang et al., 2018), were capable of generating gating pore currents when expressed in heterologous systems. On this basis, the gating pore currents were proposed to represent the common pathological mechanism linking all these mutations targeting the gating charged residues on S4 of the Nav1.5 channel (Moreau et al., 2015a; Moreau and Chahine, 2018).

It was however observed that while mutations affecting the uppermost S4 gating charges (R1 and R2) of Nav1.5 VSD I generate gating pore currents when S4 is in its resting state (that is, at hyperpolarized voltages Gosselin-Badaroudine et al., 2012a; Jiang et al., 2018), mutations affecting the more inward arginines, as R3 (R814W) on S4 of Nav1.5 VSD II, generated gating pore currents only when the S4 was in the activated state, that is, at depolarized voltages (Moreau et al., 2015b; Jiang et al., 2018). These observations cast a shadow on the overall interpretation and make the cardiac pathological consequences of gating pore currents still questionable.

Another mutation replacing a gating charge residue in the central portion of the S4 voltage sensor of Kv7.2 channel, R207W, was found to cause a syndrome characterized by neonatal convulsions (BFNC) and muscular hyperexcitability (and in turn myotonia, involuntary muscle contraction under the skin; Dedek et al., 2001). This mutation led to a marked shift of voltage-dependent activation of expressed mutant channels and a marked slowing of activation kinetics. This study also showed that lower motor neurons, thought to be involved in this syndrome, express Kv7.2 channels which would cause, in their mutant form R207W, motor neuron hyperexcitability with consequent myokymia (Dedek et al., 2001).

A similar mutation that removed the gating charge at location 207 of Kv7.2 voltage sensor, but by replacing it with glutamine instead of tryptophan (R207Q), was identified in a patient with peripheral nerve hyperexcitability and myokymia, without other neurologic symptoms (no neonatal convulsions; Wuttke et al., 2007). The milder effects of this mutation was ascribed to its smaller biophysical effects induced on the channel with regard to the voltage-dependent activation and the activation kinetics (Wuttke et al., 2007).

The few examples we briefly reviewed here to show the pathological consequences of a number of mutations involving the gating charges of the voltage sensor can be fruitfully complemented with the following more exhaustive reviews focusing on specific channels or tissues, mutation types, or pathological disorders (Cannon, 2010, 2018; Huang et al., 2017).

## Conclusions and outlook

This retrospective of the past 50 years of gating currents investigation, from their first recording in 1973, starts in fact a couple of decades earlier, with the charged gating particles that move across the membrane upon voltage changes, postulated by Hodgkin and Huxley in 1952 to explain the voltage-dependence membrane conductance to ions. Hodgkin and Huxley also proposed, as the only logical conclusion, that the movement of these charged particles should generate capacitive-like currents which, for being associated with channel gating, came to be called gating currents. It was however only 20 years later that Schneider and Chandler (1973) and Armstrong and Bezanilla (1973) first recorded the postulated gating currents. This was an extraordinary achievement since it made it possible to directly see the channels’ gating charges while moving during channel activation. Gating currents have been for all these years, and arguably stand today, the gold standard approach in the study of channel gating. Gating current measurements soon showed the many features of the gating charges behavior, namely, neither was their rising phase instantaneous nor their decay monoexponential, as expected from the original proposal of a two-state process. Further, they would get immobilized with long pulses. The amount of charge that has to move across the membrane to open a single channel could also be assessed. Fully missing was instead, at those early times, any notion of the structural counterparts of these observations.

These began to appear in the 1980s, with the cloning of the first voltage-gated channels, which disclosed their primary structures and allowed to make good guesses on their higher-level architecture, and continued, at the turn of the century, with the first x-ray crystallographic structures at atomic resolution. Several structures underlying the gating function were identified, and this allowed MD modeling techniques to come into play and give their great contribution to more thoroughly understand channel gating. Molecular dynamics also has limits, though, especially in time resolution, due to the complexity of the system studied, and output verification.

To compensate for these shortcomings, alternative strategies emerged over the years. One of these, based on Brownian dynamics for the description of the voltage sensor movements, turned out especially capable of accurately reproducing the gating currents as well as their fluctuations (Catacuzzeno and Franciolini, 2019; Catacuzzeno et al., 2021a). This occurrence appeared crucial for readdressing some channel gating issues at the atomic level, namely the so-called “shot currents” that classic experimental data on gating current noise, obtained before the channels structures were released, had suggested to be ∼2.3e_0_ (Stühmer et al., 1989; Sigg et al., 1994), in clear conflict with newer evidence suggesting shot currents of 1.0e_0_. With our Brownian model, we tested the suggestion of Crouzy and Sigworth (1993) that the high shot charge originally found could be due to the limited filter bandwidth of the experiments that would make sequential gating charges crossing the hydrophobic plug in very rapid succession appear individually indistinguishable, but lumped together, giving a higher shot charge. Analyzing a large number of simulations of a single-activating voltage sensor, we could see both elementary current shots of ∼1.0e_0_, as well as multiple events, evidence for multiple charge crossing (Catacuzzeno et al., 2021a). Moreover, the number of observed multiple crossings greatly decreased with decreasing filtering (down to ∼1.1e_0_ at 32 kHz) as anticipated by Crouzy and Sigworth (1993).

We have seen that 50 years of gating currents as the reporter of the voltage sensors movement in voltage-gated channels have given us a general picture of the dynamics of their motion and the acting forces at a fairly good level. S4 segments move in multiple steps under the traction of the field on the gating charges. We have measured the amount of motion for full activation, the charges on S4 (and around) really crucial for gating, their position with respect to the hydrophobic plug in the various VSD states, the different states of the VSD, and lately we have just started appreciating the actual trajectories of the gating charges during these operations. The next decade or two of voltage-gating investigation should go one level deeper in our understanding. We ought to be able to visualize, by integrating experiments and in silico studies, the dynamics of the single gating charges while they cross in succession the hydrophobic plug. We ought to be able to understand where, when, and how the electric forces act on them, the precise profile of the voltage drop across the hydrophobic plug, and its immediate adjacent structures, the exact succession of the interactions they make and break while passing through and the exact trajectories followed.

We do not know what techniques or approaches (some possibly not even known today) will be used to address these issues. We also do not know what part gating currents will have in it. Perhaps not much. But even then, we will always look back on them and the so many scientists who have spent a lifetime in their company with respect and gratitude for having brought us this far.

## Footnotes

1 Curiously, at the time, that value was considered an overestimation, also due to the fact that the authors had obtained in the same study a value of ∼3e_0_ with the limiting slope method (Vandenberg and Bezanilla, 1991b).

2 Although neither of the above groups tested R6, a later histidine-scanning mutagenesis study showed that R6 did not cross the transmembrane electric field upon channel activation, and so did not contribute to channel gating (Starace and Bezanilla, 2001).

3 As suggested by Crouzy and Sigworth (1993) on considering the limited filter bandwidth of recordings that would make individual charge passage at times indistinguishable, when occurring in rapid succession, and by Rodrìguez et al. (1998) on observing an increasing value of qapp with increasing depolarization.

4 Based on the cryo-EM structures of the human BK channel (Tao and Mackinnon, 2019).

5 As of a survey in 2010, 21 of a total of 53 missense mutations reported in Nav1.4, and 6 of 7 mutations in Cav1.1 were in the S4 voltage sensor (Cannon, 2010).
